# Why healthy eating is not equally attainable for all: a participatory systems perspective on dietary behaviour of people living on a low income in the Netherlands, and recommendations for systemic change

**DOI:** 10.1186/s13690-026-01838-x

**Published:** 2026-01-28

**Authors:** Sanne K. Djojosoeparto, Nieke A. Sonneveld, Carlijn B. M. Kamphuis, Maartje P. Poelman

**Affiliations:** 1 Chair Group Consumption and Healthy Lifestyles, Department of Social Sciences, Wageningen University, Wageningen, The Netherlands; 2 Department of Interdisciplinary Social Science, Utrecht University, Utrecht, The Netherlands

**Keywords:** (un)healthy diets, Low income, Disadvantaged socioeconomic positions, Participatory systems dynamic approach, Leverage points, Actions for systemic change, Perspectives of professionals and citizens

## Abstract

**Background:**

Living on a low income is associated with multiple interacting factors and mechanisms that contribute to less healthy diets. Yet research has rarely examined these systemic drivers in depth among people on low incomes, including the perspectives of both citizens with experiential knowledge as well as professionals. Therefore, this study aimed to gain insights into the complex system dynamics underlying dietary behaviour of people on a low income, from the perspective of both citizens with experiential knowledge and professionals.

**Methods:**

This study applied a participatory, system-based approach. Three group model building sessions were conducted in a Dutch municipality (November 2024); one with citizens with experiential knowledge (*n* = 15); one with professionals with expertise in the field of food, nutrition, health, social support (e.g., financial, family), involved in policies or working directly with people living on a low income and/or those facing diet/health-related issues (*n* = 13), and one combined session with both groups. During the first two sessions, citizens and professionals created separate Causal Loop Diagrams (CLDs) including factors and mechanisms influencing dietary behaviour. During the third session, citizens and professionals identified leverage points for change and corresponding actions to facilitate healthier dietary behaviour for people on a low income. The research team merged the separate CLDs and actions proposed into one integrated CLD and action list, after the sessions.

**Results:**

The perspectives of citizens with experiential knowledge and professionals overlapped and complemented each other well, leading to an integrated CLD with 62 factors. Seven subthemes were identified: (1) Government and policy, (2) Food industry, (3) Social and societal factors, (4) Food environment, (5) Resources and daily living conditions, (6) Mental and physical wellbeing, and (7) Individual behaviour and characteristics. The CLD revealed three core dynamics underlying dietary behaviour of people on a low income: scarcity and reduced wellbeing, the unhealthy food environment, and social and societal barriers. Citizens and professionals proposed 30 actions addressing these dynamics for systemic change.

**Conclusions:**

This study yielded insights into the complex system that complicates healthy eating for people on a low income, based on the perspectives of citizens and professionals. Actions at different system levels are needed to facilitate healthy dietary behaviour among people on a low income. In this, it is essential to involve professionals as well as citizens with experiential knowledge in the development of policy and support services.

**Supplementary Information:**

The online version contains supplementary material available at https://doi.org/10.1186/s13690-026-01838-x.

**Table:** 

Text box 1. Contributions to literature
• The results provide in-depth insights into the complex system including different core dynamics (i.e., scarcity and wellbeing, the food environment, and social/societal environment) underlying dietary behaviour of people on a low income.
• The results imply that including lived and professional experiences in research and policy is essential, to increase mutual understanding and to ensure that the actions are well aligned with lived realities.
• The results suggest 30 actions for systemic change, substantiating evidence that actions at different system levels are needed to ensure greater impact, as there is no single solution to break through the current system.

## Background

Socioeconomic inequalities in health and diet are observed and increasing within many high-income countries [1, 2], including the Netherlands [3]. Overall, a higher risk of developing overweight, obesity and diet-related chronic diseases (such as cardiovascular diseases and type 2 diabetes) is associated with living in more disadvantaged socioeconomic conditions, including living on a low income, and having limited access to education, housing, social support, and opportunities (e.g., unemployment) [2, 4, 5]. For example, in the Netherlands, overweight and obesity are generally less prevalent among individuals in higher income groups than among lower income groups [6]. Unhealthy diets are contributing to the high prevalence rates of overweight, obesity and diet-related chronic diseases [7]. Although most people in the Netherlands do not eat in accordance with the national dietary guidelines [8], higher consumption of ultra-processed foods rich in saturated fat, sugar and salt (such as snacks, soft drinks), and lower consumption of fresh unprocessed products (such as vegetables and fruit) are more often associated with more disadvantaged socioeconomic positions than with more advantaged ones [9–11].

Living on a low income, which is a key aspect of socioeconomic disadvantage, may contribute via various factors (e.g., societal, commercial, political) and mechanisms to unhealthier dietary behaviour [12]. A theoretical framework that addresses the impact of these factors and mechanisms on dietary behaviour and dietary inequalities is the Nutrition Equity Framework (NEF) [13]. This framework illustrates how structural determinants, such as sociopolitical contexts (e.g., societal ideas, institutions, actors and interests) and social stratification (i.e., socioeconomic position, capital, and potential) shape intermediate determinants, including daily living conditions and food, care and health environments, thereby impacting nutrition inequity [13]. However, a possible concern is that the NEF may be interpreted or applied in a manner that assumes linearity, which may not reflect the complexity of interactions between and dynamics among factors influencing dietary behaviour and inequalities.

To address complex public health problems, systems science is receiving growing attention, conceptualising these problems as outcomes of dynamic systems, constituted by interconnected factors operating at various levels [14]. A complex system consists of elements, interconnections, and a function or purpose, where changes in one part can trigger intended or unintended consequences in other parts of the system over time [15, 16]. In public health, systems thinking is commonly operationalised using systems dynamic approaches to provide insights into the underlying dynamics influencing public health problems, and to collaboratively identify actions (e.g., policies and interventions) tackling determinants of these problems that would be most effective [14, 17–19]. While these approaches have been increasingly used to understand the complex systems of obesity among disadvantaged socioeconomic groups, youth or in general [16, 20–22], healthy diets in general [18, 23] or food environments experienced by (disadvantaged socioeconomic) communities [17, 24, 25], few studies have explicitly applied such approaches to understand the systemic drivers and dynamics of dietary behaviour among people on a low income and to identify leverage points and actions for change to facilitate healthy dietary behaviour.

Moreover, few systems-based studies have applied a participatory approach integrating lived and professional experiences to gain insights into this complex system. However, this is important because these provide valuable insights into the underlying drivers and mechanisms of dietary behaviour, which may remain invisible through other research methods [26, 27]. Moreover, actively including citizens and professionals can increase the quality, societal relevance and applicability of study findings and recommendations for change [28]. Participatory methods can therefore contribute to connecting and mutual understanding of each other’s perspectives, citizens from their living world perspective and professionals from their institutional or policy perspective [27]. This is essential for developing effective policy strategies and interventions that align with the needs of people the interventions are aimed at [29]. Local governments play a key role in enabling inclusive approaches, as they are crucial actors in system-based strategies to address nutrition inequity due to their proximity to disadvantaged communities and ability to engage local stakeholders and citizens [30]. To explore this in practice, the present study was conducted in a disadvantaged neighbourhood in a municipality in the Netherlands, in dialogue with municipal officials, particularly concerning the organisation of the study and stakeholder access.

Addressing the above mentioned knowledge gaps, this study aims to:

1. Gain insights into the complex system of factors and their interconnections and dynamics that underly dietary behaviour of people on a low income, from the perspective of citizens with experiential knowledge and professionals.
2. Identify leverage points for change in the system and corresponding actions to facilitate healthier dietary behaviour for people on a low income, from the perspective of both citizens with experiential knowledge and professionals.

## Methods

### Study setting

The study was conducted in Veldhuizen, a neighbourhood in the municipality of Ede in the Netherlands. In 2025, Ede had a population of 124.214 residents and the Veldhuizen neighborhood had a population of 14,585 residents [31, 32]. This neighbourhood was selected because, compared to other neighbourhoods, Veldhuizen is characterized by a relatively high number of households on a low income [33]. In Veldhuizen, 7% of households had to live on the social minimum or lower in 2023, 12,3% of households had an income lower than 120%^1^ of the social minimum in 2023 and 9 out of 100 citizens received benefits [34]. Moreover, according to the most recent figures, in 2020 the prevalence of people with overweight (54.2% vs. 50.0%) and obesity (18.1% vs. 13.0%) was higher in Veldhuizen compared to average for the entire municipality of Ede [33, 34].

The food environment of the Veldhuizen neighbourhood in Ede is primarily concentrated in the Bellestein shopping centre. The area includes one large-chain supermarket, as well as two privately owned supermarkets, consisting of a local neighborhood grocery store and an international supermarket specializing in multicultural and halal products. In addition, the shopping centre hosts several quick service and fast food outlets, including a snackbar, a lunchroom, pizzeria take-away, doner-kebab eatery, Chinese restaurant and sushi take-away.

### Study design

Group Model Building (GMB), a participatory system approach, was used to capture diverse stakeholder perspectives and knowledge [35]. GMB supports participants in visualizing their mental models, e.g. their beliefs and assumptions about the functioning of a complex system [16, 36]. Three in-person GMB sessions were held in November 2024; one held with citizens in the Kernhuis community centre of Veldhuizen, one with professionals at the municipality centre of Ede, and one combined session with both groups in the Kernhuis community centre of Veldhuizen. All participants were 18 years or older and provided written informed consent for the study.

### Participant recruitment and study procedure

#### Participant recruitment for the GMB sessions

Purposive sampling was used to recruit professionals working with people living on a low income and/or people with diet/health-related issues in the municipality of Ede. Professionals with diverse expertise in the field of food, nutrition, health, social support (e.g., financial, family), and having diverse positions were approached (e.g. policymaker, social worker, financial counsellor, dietitian, food bank volunteer, general practitioner, employment support officer). Lists of relevant professionals to invite were gathered through contact with a local food policy official and a community health coordinator. To further reach relevant professionals, we additionally applied snowball sampling, asking professionals to refer colleagues within their professional networks. E-mail invitations were sent to professionals, with the request to forward the invitation to other professionals who may have valuable knowledge and expertise. Moreover, we informed and recruited professionals during several meetings in the municipality, i.e., a meeting with the community manager of Veldhuizen and food policy official in Ede (June 2024), a coordination meeting with professionals working in the neighbourhood of Veldhuizen (September 2024), and a meeting with the social team of Ede (September 2024). In total, 41 professionals were directly invited by us via e-mail, and more professionals were invited through contacted referrals from those directly contacted. The information regarding professionals’ job descriptions was gathered during the above mentioned recruitment process. We specifically approached individuals because of the roles they held, and in some cases, we were informed of their positions through the above mentioned contacts. Participating professionals received a financial compensation (20 EUR gift voucher) for participating in one or two sessions.

To recruit citizens from the neighbourhood Veldhuizen in Ede, we distributed a flyer with information about our study, inviting people who had experience with making ends meet on a low income to participate *(“… If you have to live on a low income, it can be even harder to make healthy food choices. Do you have experience with this? …”)* Previously approached professionals were asked to share this information flyer with any citizens in their network experiencing financial insecurity. Furthermore, the flyer was distributed at several locations in and around Veldhuizen, such as community centres, several schools, the library, supermarkets, dentist, and the medical centre. Additionally, a volunteer at the food bank put the flyers in food parcels of citizens of Veldhuizen. Citizens received a financial compensation (50 EUR gift voucher per session) for participating in our study.

Eventually, thirteen professionals with diverse expertise in the field of food, nutrition, health, and social support (e.g., financial, family), involved in policies or working directly with people living on a low income and/or those facing diet/health-related issues in the municipality of Ede (hereafter ‘professionals’), and fifteen citizens from the Veldhuizen neighbourhood in Ede (hereafter ‘citizens’) participated in our study. Table 1 includes a description of the various roles or characteristics of these participants.

#### GMB sessions

Three GMB sessions of three hours each were conducted. Protocols were developed, based on existing GMB scripts, for the sessions with professionals and citizens [37] (Additional file 1). Per session, six to eight researchers were present to take notes and facilitate discussions. All sessions were audio-recorded.

The aim of the first two sessions was to identify systemic factors and mechanisms underlying (un)healthy dietary behaviour of people on a low income, from the perspective of professionals and from the perspective of citizens. After a short introduction by SD about (un)healthy eating patterns, influences on dietary inequalities, and system approaches, participants first individually identified and wrote down factors that could influence healthy eating for people on a low income. To help participants consider determinants of dietary behaviour on multiple levels (e.g., structural, environmental) a simplified version of the NEF was introduced and a large visual version of the simplified NEF was placed on each table. Facilitators asked participants to identify factors that influence dietary behaviour, keeping the different levels of the NEF in mind while being free to introduce additional factors beyond the framework. The participants then discussed these factors in groups of about three people facilitated by a researcher (SD, NS, MP, CK and other researchers) and selected and wrote down the ten most important factors per group. Facilitators actively ensured that all participants had the opportunity to speak, preventing any single participant from dominating the discussion and encouraged participants to elaborate on their ideas. The ten most important factors were then discussed in plenary with all participants, after which it was discussed how these most important factors are related to each other and influence each other. Based on participants input, MP and CK physically built a system map on the wall in the form of a ‘Causal Loop Diagram’ (CLD), visualizing the interactions between the factors that influence (un)healthy eating behaviour of people on a low income. A CLD is a tool used in system thinking which includes the factors, connections between the factors and feedback loops that help to gain insights into how a system behaves [16, 35, 38]. NS simultaneously built a digital version of the CLD using STICKE (Systems Thinking In Community Knowledge Exchange) software [39], which was displayed to participants at the end of the session for a clearer and more accessible representation of the system map. At the end of the sessions background questionnaires were provided to participants with questions about their personal characteristics, such as age, gender, and participants ethnic background.

After these sessions, the research team further refined the list of factors, including participants’ explanations, and the CLDs of the professionals and citizens. All data sources generated during the GMB sessions, including audio-recordings, written materials, and facilitator notes were reviewed to verify, complete, and consolidate the factors and connections proposed by participants, ensuring that no contributions were overlooked and that ambiguous or overlapping formulations were clarified. Next, we developed an integrated list of factors and their connections by merging similar items and noting whether a factor had been proposed by professionals, by citizens, or by both groups. The first versions of the CLDs developed through STICKE, were replicated and based on the review of all GMB data sources further refined in KUMU, a software tool to visualize and map systems and relationships [40]. We used KUMU to display all factors identified in the sessions and to map the connections between them.

The third session was a combined session with professionals and citizens. The aim of this session was to identify leverage points in the system and propose actions to facilitate healthy dietary behaviour for people on a low income. Citizens and professionals were first split up in separate groups and were asked to identify, based on their developed CLD, around five key factors that they believed required action (so-called places to intervene or leverage points [15, 41]. At this stage, participants also had the opportunity to make final adjustments to their CLD, such as adding factors and connections. Thereafter, professionals and citizens formulated actions based on the leverage points to facilitate healthy dietary behaviour for people living on a low income. This process started individually, and later, participants shared their actions with each other in groups of about three people, facilitated by researchers, while citizens and professionals continued working in separate groups. Then, each group identified the three most important actions and shared these with the rest of the group. Afterwards, the professionals and citizens came back together. One by one, a citizen and a professional presented their most important leverage points and actions to the entire group, explaining how these leverage points and actions relate to the system. Finally, there was an opportunity to ask questions about each other's results and reflect on each other's CLDs and formulated actions.

#### CLD refinement and integration

After the third session, SD and NS further refined the list of factors and the CLDs (e.g. merging overlapping factors, adding factors and connections), based on the final adjustments made by participants in the final GMB session, reviewing data generated including audio-recordings, written material collected and notes taken during the sessions. To verify whether the refined results still aligned with the ideas of the participants, they were contacted via email and given the opportunity to submit any corrections and/or suggestions. The research team then merged the two separate CLDs from citizens and professionals into one integrated CLD. This was done by first integrating similar factors and connections, and then adding factors and connections addressed by one of the two groups. In the list of factors and the CLD, it is indicated which factors were identified by citizens, professionals or both groups. Based on this, we identified the similarities and differences in factors, proposed by professionals and citizens.

#### Identification of subthemes and dynamics

This merged CLD was then analysed by the research team (SD, NS, MP and CK) by identifying subthemes, feedback loops and key dynamics. The subthemes refer to groups of factors that describe similar topics and influences in the system. We distinguished in our analysis between the system’s structure, comprising factors, interconnections, and feedback loops, and its functioning, which refers to the underlying dynamics and their significance [16, 42, 43]. Feedback loops were reviewed but not analysed in this study, instead the analysis focused on key dynamics underlying the system. This was done because key dynamics include the most striking mechanisms and drivers that maintain the system, in this case, the dynamics that sustain (un)healthy dietary behaviour of people on a low income. Identifying key dynamics enables highlighting structural patterns and drivers of the system that influence how the system behaves, without requiring full closure of each feedback loop [15, 43, 44]. Moreover, although the feedback loops were used to identify key patterns, it was not feasible to analyse all feedback loops (8610) that were automatically detected by Kumu in our CLD. To identify the key dynamics, the CLD in Kumu was analysed by the research team, by: (1) identifying key patterns that spanned and connected different subthemes, (2) analysing how the separate subtheme mechanisms feed into each other, and (3) considering the leverage points identified by citizens and professionals [16, 43].

#### Refinement, integration and analysis of the actions for systemic change

The research team further refined the list of actions that came out of the third session, using the same systemic process applied to the factors, drawing on the audio-recordings, written material collected, and notes taken during the sessions. Also, we created an integrated list of actions, and analysed which actions were proposed by professionals, by citizens or by both groups. The refined action list was shared via email with participants to provide them with the opportunity to share any corrections and/or suggestions. Based on this verification a final action list was compiled. As the content of the formulated actions closely relates to the dynamics identified in the CLD, the researchers divided the action list accordingly. To explore which levels of the system were targeted by the proposed actions, the research team analysed the actions using the Action Scales Model [44]. The Action Scales Model describes four levels to intervene within a system, with deeper levels (i.e., beliefs and goals levels) providing greater potential for changing how the system functions [44]. *Events* relate to the observable issues (behaviours and proxy outcomes) and are symptoms of the system. *Structures* relate to the patterns, relationships, information flows and physical structures that cause events to occur. *Goals* refer to the ambitions or targets that the system (or parts within the system) are working towards. Finally, it is the *beliefs*, norms, values and attitudes of systems architects (i.e. those who influence the structure and workings of a system) that cause the system to function as it does. Analysing which different systems levels are targeted by the actions can help to assess the depth and potential impact of the actions [44].

## Results

### Study participants

In total thirteen professionals with expertise in the field of food, nutrition, health, support provision (e.g. financial/food/family support), involved in policies or working directly with people living on a low income and/or people with diet/health-related issues, participated in our study. The professionals had a mean age of 44 (range 25- 67 years), and ten professionals identified as female and three as male. Fifteen citizens from the Veldhuizen neighbourhood in Ede participated in our study. Citizens had a mean age of 54 (range 31–83 years), and 12 citizens identified as female, two as male, and one citizen as other. Table 1 includes a description of the various roles or characteristics of these participants and gives an indication of the GMB session(s) the participant attended.

**Table 1:** Characteristics of participants of the Group Model Building (GMB) sessions

	**Gender**^**a**^	**Role of participant**	**Ethnic background**^**b**^	**Attendance GMB session professionals**^**c**^	**Attendance GMB session citizens**^**c**^	**Attendance GMB session professionals + citizens**^**c**^
1	F	Professional; Policy official health	Dutch	X		X
2	F	Professional; Health coordinator	Dutch	X		X
3	F	Professional; Policy official health	Dutch	X		X
4	F	Professional; Youth health promotion officer	Dutch	X		X
5	F	Professional; Dietician	Dutch	X		X
6	M	Professional; Family support worker	Dutch	X		X
7	F	Professional; Policy official food	Dutch	X		
8	M	Professional; Community manager	.	X		
9	F	Professional; Social worker	Dutch	X		
10	F	Professional; Social worker	Dutch	X		
11	F	Professional; Volunteer food bank	Dutch	X		
12	F	Professional; Financial support worker	Dutch			X
13	M	Professional; Social worker/coordinator	Non-western			X
14	F	Citizen with experiential knowledge	Dutch		X	X
15	F	Citizen with experiential knowledge	Dutch		X	X
16	F	Citizen with experiential knowledge	Dutch		X	X
17	F	Citizen with experiential knowledge	Dutch		X	X
18	M	Citizen with experiential knowledge	Dutch		X	X
19	F	Citizen with experiential knowledge	Dutch		X	X
20	F	Citizen with experiential knowledge	Dutch		X	X
21	F	Citizen with experiential knowledge	Dutch		X	X
22	M	Citizen with experiential knowledge	Dutch		X	X
23	F	Citizen with experiential knowledge	Dutch		X	X
24	F	Citizen with experiential knowledge	Dutch		X	X
25	F	Citizen with experiential knowledge	Dutch		X	X
26	Other	Citizen with experiential knowledge	Non-western		X	X
27	F	Citizen with experiential knowledge	Non-western		X	X
28	F	Citizen with experiential knowledge	Dutch			X

Study: Why healthy eating is not equally attainable for all: A participatory systems perspective on dietary behaviour of people living on a low income in the Netherlands, and recommendations for systemic change, Ede the Netherlands, 2024–2025

^a^’F’ = Female and ‘M’ = Male

^b^Answer categories were: Dutch; Western migration background (e.g., European, North American, Australian); Non-Western migration background (e.g., Turkish, Moroccan, Surinamese, Antillean, Indonesian); Other, namely; Prefer not to say

^c^’X’ means ‘was present at this GMB session’

### CLD and subthemes

**Fig. 1:**
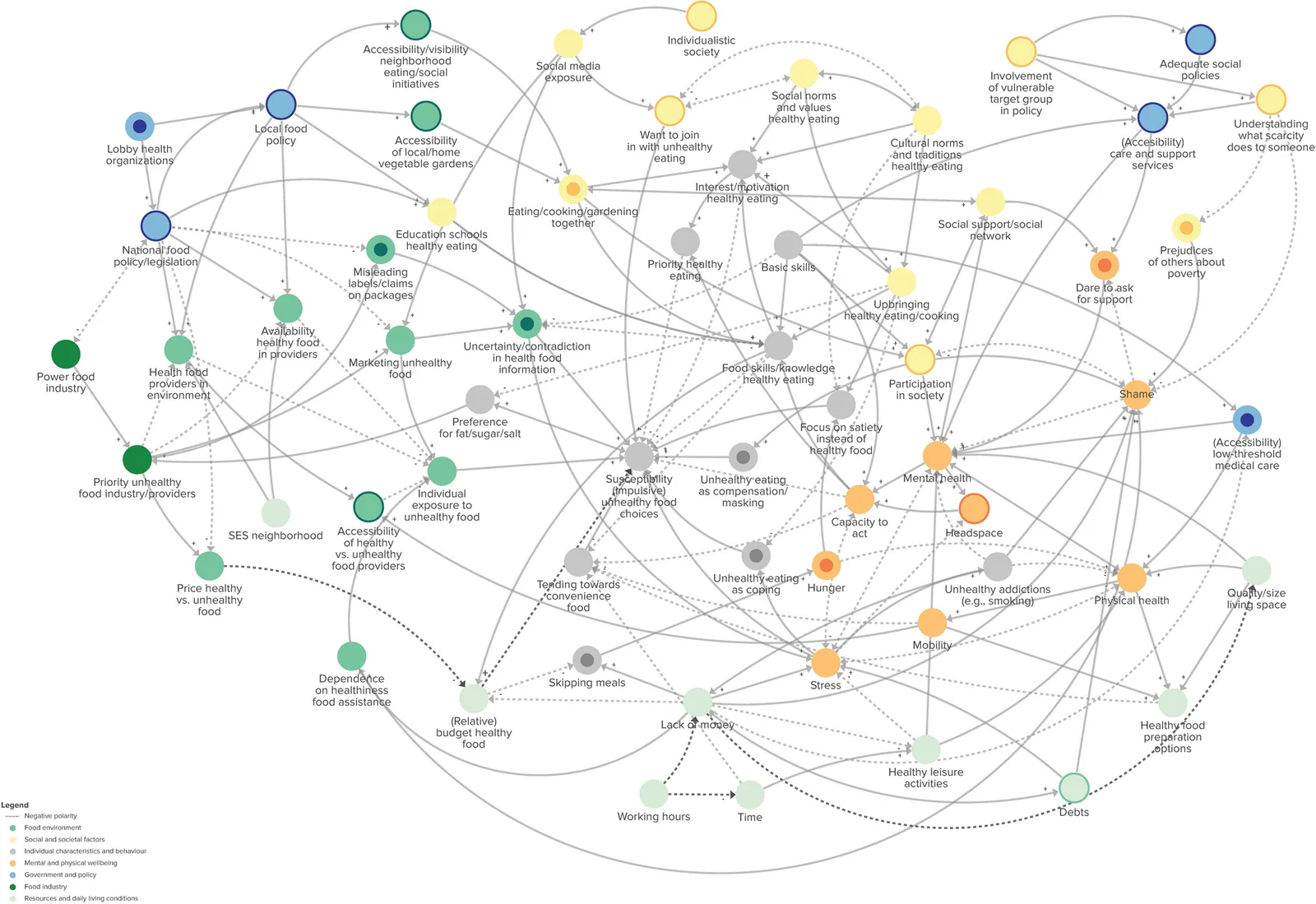
Integrated CLD on (un)healthy dietary behaviour of people who have to make ends meet on a low income. *bullseye means factor only proposed by citizens; border means factor proposed by professionals; plain circle means factor proposed by citizens as well as professionals; + means positive polarity (an increase in factor A leads to an increase in factor B); - means negative polarity (an increase in factor A leads to a decrease in factor B). (Study: Why healthy eating is not equally attainable for all: A participatory systems perspective on dietary behaviour of people living on a low income in the Netherlands, and recommendations for systemic change, Ede the Netherlands, 2024–2025)

Figure 1 illustrates the full CLD, showing the interconnected structure of 62 factors. This figure also visualizes how these factors cluster into seven subthemes: (1) Government and policy, (2) Food industry, (3) Social and societal factors, (4) Food environment, (5) Resources and daily living conditions, (6) Mental and physical wellbeing, and (7) Individual behaviour and characteristics.

#### Government and policy

Refers to the role of public governance in shaping conditions for healthy eating among low-income populations. It includes food environment policies at local and national levels, lobbying efforts that influence policy implementation, and broader social policies and regulations. The theme also covers the organisation and accessibility of care and support services, such as affordability, findability, and low-threshold medical services.

#### The food industry

Addresses the structural influence and market power of the food industry in shaping the food environment and affecting policy and legislation. It encompasses industry’s prioritisation of profit over health, often leading to a predominance of unhealthy products and a food environment that makes healthy choices more difficult.

#### Social and societal factors

Encompasses influences from social networks, cultural practices, upbringing, and education that shape dietary norms and habits. It also includes social support and participation, communal activities (e.g., eating/cooking/gardening together), and broader societal dynamics (e.g., individualisation, social media). Additionally, it considers stigma and perceptions around poverty, understanding the impacts of living in scarcity, and the importance of including vulnerable groups as experts by experience in policy development, each affecting mental wellbeing and access to support.

#### The food environment

Focuses on the availability, accessibility, price, and marketing of foods. It includes the healthfulness of food providers, the relative accessibility of healthy vs. unhealthy outlets, prices of healthy vs. unhealthy options, marketing and labelling practices (including misleading claims), and neighborhood initiatives (e.g., communal cooking or local gardens). Together, these factors shape individual exposure to unhealthy foods.

#### Resources and daily living conditions

Covers material resources and everyday constraints that affect dietary behaviour such as lack of money, debts, housing conditions, neighborhood socioeconomic status, time scarcity, long working hours to make ends meet, and limited opportunities for healthy leisure activities.

#### Mental and physical wellbeing

Addresses how physical health, mobility, hunger, and mental health (e.g., stress, shame) influence cognitive bandwidth (headspace) and the ability to act (e.g., plan, shop, cook, and choose healthy foods). It highlights the way reduced wellbeing can hinder prioritising healthy food choices and seeking for support.

#### Individual characteristics and behaviour

Captures skills, knowledge, motivation, and preferences that guide dietary behaviour. It includes basic and food-related skills, interest in and prioritisation of healthy eating, a focus on satiety over health under scarcity and hunger, reliance on convenience foods, and coping behaviours such as unhealthy eating or addictions (e.g., smoking, alcohol). 

Most factors identified were mentioned by professionals as well as citizens (Fig. 1 and Additional file 2). However, the most notable differences can be observed in the ‘Government and policy’ subtheme, where none of the factors within this domain were jointly identified by both professionals and citizens. In this subtheme, professionals mentioned factors related to food and social policies, whereas citizens mentioned factors related lobbying by health organisations and (the accessibility of) low-threshold medical care. In the other subthemes, many factors were brought forward by both groups. Some factors, however, were specifically highlighted by either citizens or professionals, although these were often similar in nature.

For example, in the subtheme ‘social and societal factors’, citizens mentioned prejudices about poverty, whereas professionals noted the factor understanding the impact of living in scarcity. The description of the factors in the subthemes, including the similarities and differences observed in identified factors between citizens and professionals can be found in Additional file 2. A full list of factors including an explanation and the connections per factor can be found in Additional file 3.

### Key dynamics

**Fig. 2:**
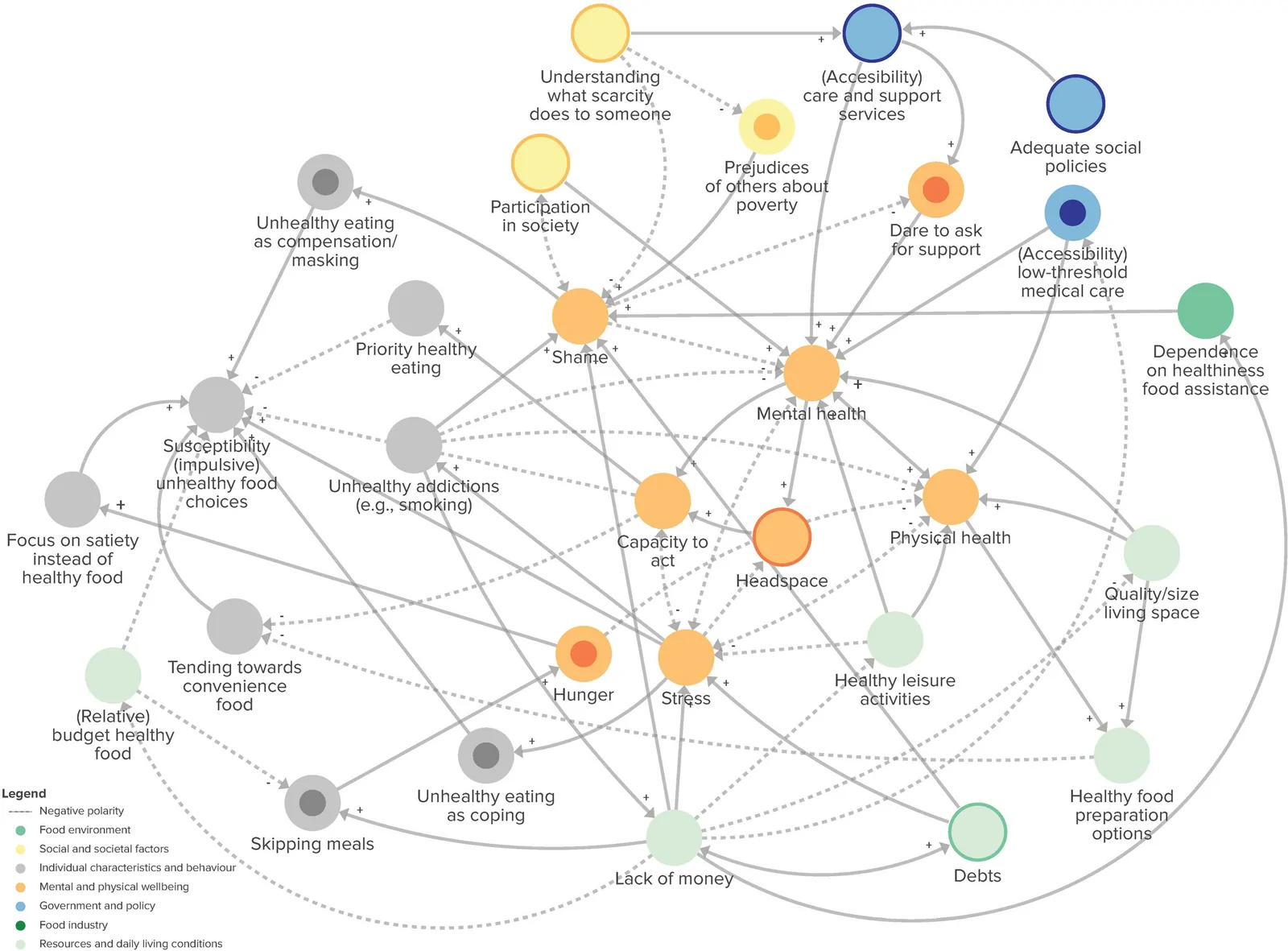
Dynamic 1 - Scarcity and wellbeing. *bullseye means factor only proposed by citizens; border means factor proposed by professionals; plain circle means factor proposed by citizens as well as professionals; ; + means positive polarity (an increase in factor A leads to an increase in factor B); - means negative polarity (an increase in factor A leads to a decrease in factor B). (Study: Why healthy eating is not equally attainable for all: A participatory systems perspective on dietary behaviour of people living on a low income in the Netherlands, and recommendations for systemic change, Ede the Netherlands, 2024-2025)

**Fig. 3:**
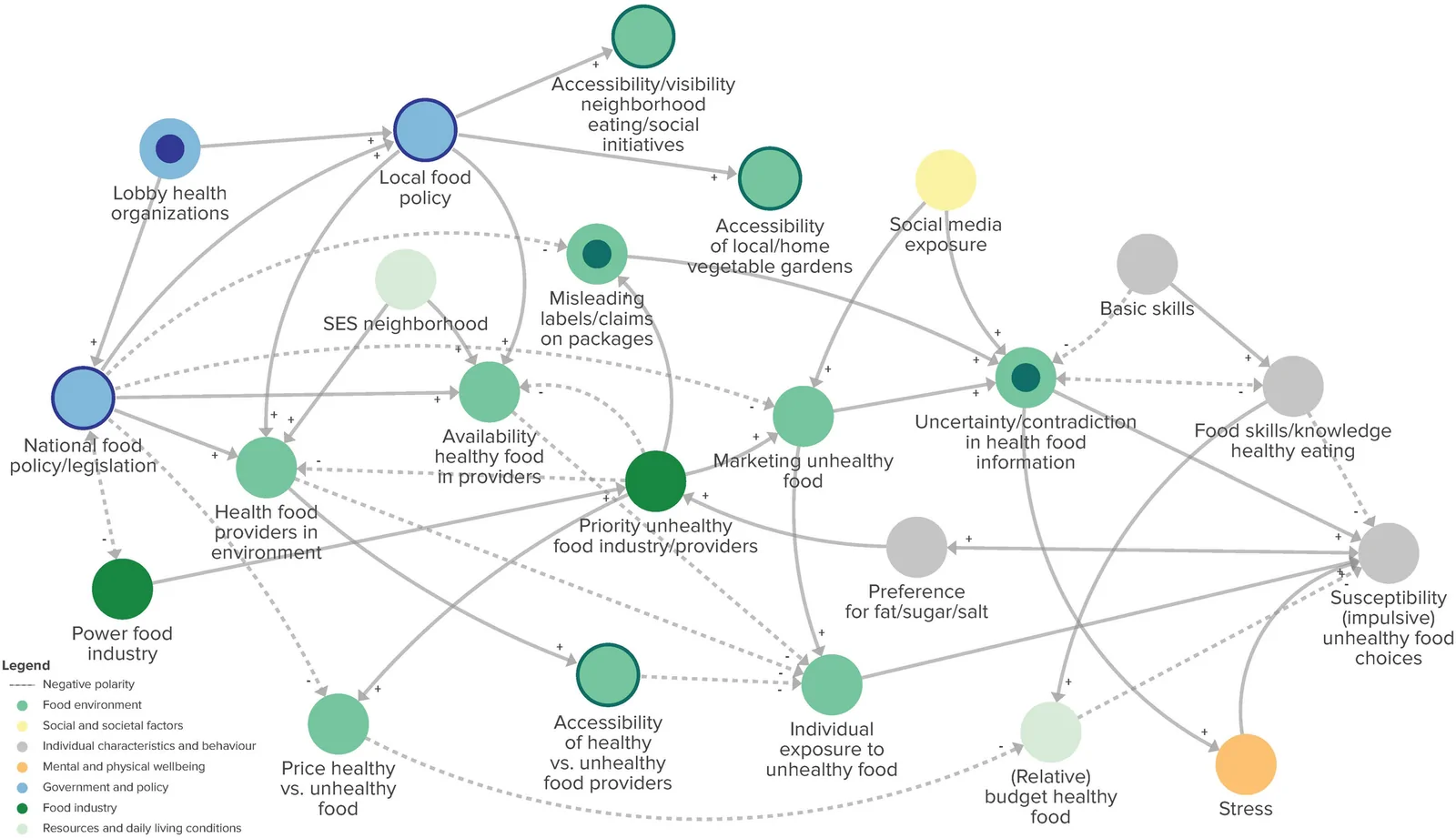
Dynamic 2 - Food environment. *bullseye means factor only proposed by citizens; border means factor proposed by professionals; plain circle means factor proposed by citizens as well as professionals; ; + means positive polarity (an increase in factor A leads to an increase in factor B); - means negative polarity (an increase in factor A leads to a decrease in factor B). (Study: Why healthy eating is not equally attainable for all: A participatory systems perspective on dietary behaviour of people living on a low income in the Netherlands, and recommendations for systemic change, Ede the Netherlands, 2024-2025)

**Fig. 4:**
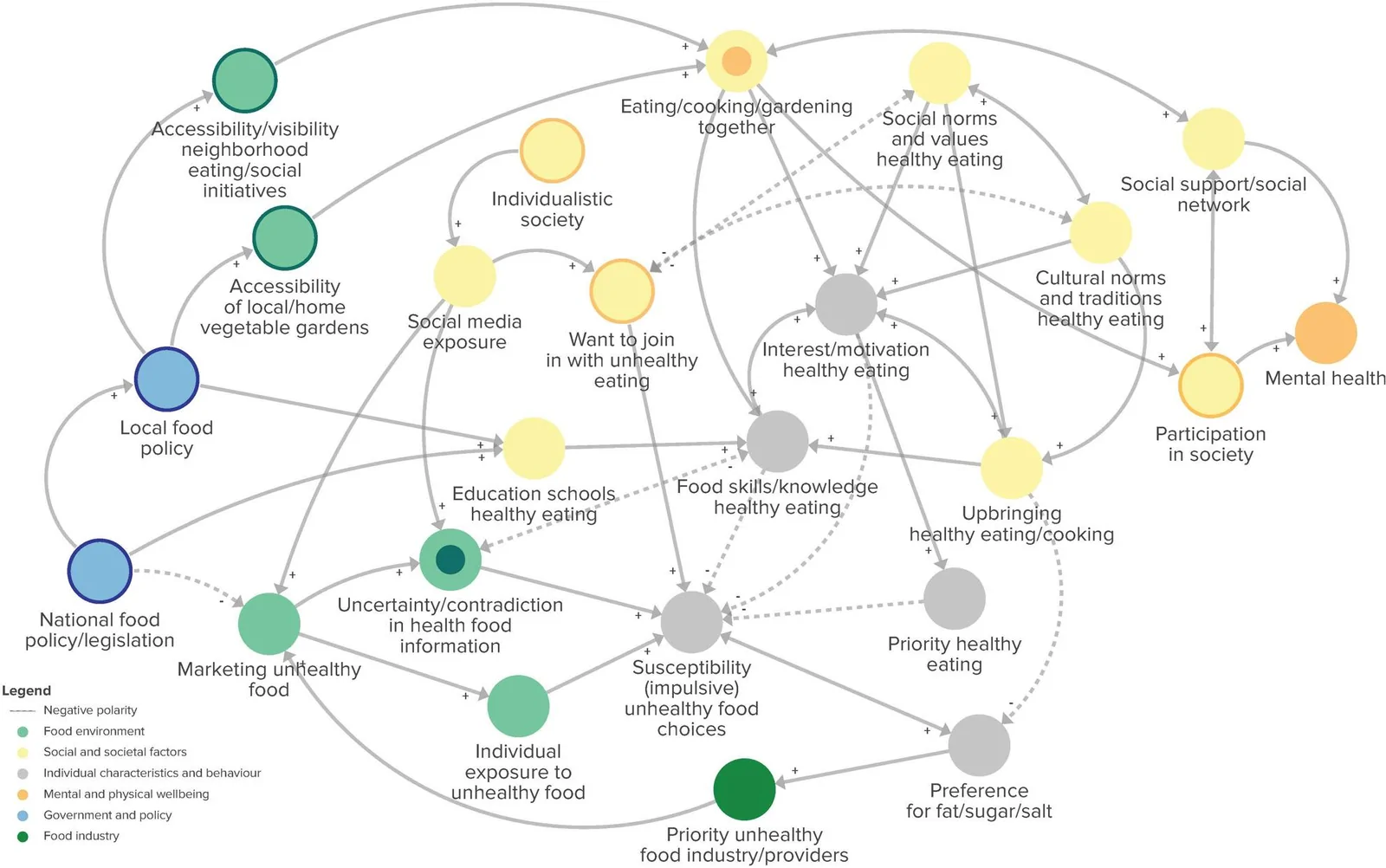
Dynamic 3 - Social and societal environment. *bullseye means factor only proposed by citizens; border means factor proposed by professionals; plain circle means factor proposed by citizens as well as professionals; ; + means positive polarity (an increase in factor A leads to an increase in factor B); - means negative polarity (an increase in factor A leads to a decrease in factor B). (Study: Why healthy eating is not equally attainable for all: A participatory systems perspective on dietary behaviour of people living on a low income in the Netherlands, and recommendations for systemic change, Ede the Netherlands, 2024-2025)

Based on the CLD, including the factors, connections and feedback loops, and the identified leverage points three key dynamics have been identified, including (1) Scarcity and wellbeing, (2) Food environment, and (3) Social and societal environment. Each dynamic integrates elements from multiple subthemes, with some subthemes playing a more prominent role than others. These interconnections are illustrated in Figs. 2, 3 and 4, which show how factors from different subthemes collectively contribute to each dynamic. Figure 2 visualises the first key dynamic, *Scarcity and wellbeing*. This part of the CLD illustrates how financial strain, stress, and reduced mental wellbeing interact and influence dietary behaviour of people living on a low income, Fig. 3 presents the second key dynamic, the *Food environment*. It shows how the unhealthy food environment makes it difficult to eat healthily, especially for people with limited financial resources. Figure 4 depicts the third key dynamic, *Social and societal environment*, highlighting the influence of factors such as upbringing, socio-cultural eating habits, and the influence of social media.

The following sections describe in more detail how each dynamic contributes to maintaining the current system, which complicates healthy eating for people living on a low income, based on participants’ perspectives.

#### Dynamic 1- scarcity and wellbeing

During the sessions, participants identified various ways in which living on a low income may impact dietary behaviour via scarcity and wellbeing. On the one hand, participants described how having a lack of money can lead to insufficient budget for healthy food, leading to skipping meals and feelings of hunger, making satiety instead of health a primary concern. Furthermore, participants pointed out that a lack of money may result in living in unfavorable living conditions, with limited options for preparing healthy food. This makes people susceptible to unhealthy food choices that are relatively cheap and easy to prepare.

On the other hand, participants identified that a lack of money and/or having debts can cause feelings of shame and stress, for instance when becoming dependent on the food bank, which may result in poorer mental health and less participation in society. In addition, lack of money was perceived as a barrier to participate in healthy leisure activities, which can further negatively affect mental and physical health.

Furthermore, participants indicated that there is often little understanding of what it is like to live in scarcity and what impact this can have on someone's daily life in today's society and among professionals. As a result, policy and support services do not always match the needs of people who have to make ends meet on a low income. In addition, it was noted that misconceptions and prejudices about poverty can lead to shame and not daring to ask for support, increasing mental problems.

Participants described that mental and physical wellbeing influence dietary behaviour in various ways. Experiencing stress or poor mental health can lead to less thinking space and ability to act, for example, for making conscious healthy food choices. In addition, it was mentioned that people can start eating unhealthy as a direct reaction to shame and stress, for example as a way to mask/compensate for poverty, or to deal with stress (coping mechanism). Participants indicated that stress not only has consequences for unhealthy eating, but can also lead to unhealthy addictions, such as smoking or alcohol use, further worsening financial problems (lack of money) and creating a vicious circle.

#### Dynamic 2- food environment

Participants identified various ways in which the current food environment create barriers to healthy eating, especially for people on a low income. First of all, participants noted that people on a low income are generally more exposed to unhealthy foods, because there are relatively more unhealthy food providers and a larger presence of unhealthy food in providers in neighborhoods with a low socio-economic status (SES) than in neighborhoods with a high SES. Second, participants mentioned that the abundance of unhealthy food marketing (on the street, in shops, online and on packaging) increases exposure to unhealthy foods, particularly among low income groups.

Third, participants described that the abundance and contradictory information about the healthiness of foods, spread through misleading claims or social media influencers, creates confusion about what is healthy or unhealthy. This can lead to stress, especially for people who have less basic or food skills. Finally, it was indicated that the prices of healthy food are higher compared to unhealthy food, leaving those with little money and/or limited food skills/knowledge with (relatively) less budget to eat healthily.

Susceptibility of people with little money to unhealthy food choices, can increase the chance of developing a preference for unhealthy foodsand. affects the demand for unhealthy foods. This causes the food industry to prioritise the production and sale of these products, contributing to the large supply, extensive marketing and relatively low prices of unhealthy foods.

Participants described that the absence of governmental legislation and regulations maintains this unhealthy food environment and the power the food industry has.

#### Dynamic 3- social and societal environment

Participants identified various ways in which the social and societal environment creates barriers to healthy eating and ultimately increases susceptibility to unhealthy food choices, especially for people who have to make ends meet on a low income. It was indicated that unhealthy eating behaviour in someone's social-cultural network, such as with family, friends or social media (e.g., influencers) can stimulate unhealthy eating habits (e.g., to want to join in unhealthy eating). This can also reduce the attention to healthy eating in upbringing, resulting in a lower interest or motivation, and therefore priority to eat healthily in daily life. At the same time, participants indicated that the increasingly individualistic society amplifies the use and influence of social media, increasing individual exposure to unhealthy food promotions and reinforcing unhealthy eating habits.

Participants mentioned that certain healthy eating initiatives within the social environment (e.g., eating, cooking or gardening together) are important because they can contribute to improving food skills, stimulating interest in healthy eating, participating in society and developing a social network, the latter two particularly important for mental health. However, participants indicated that food education or initiatives to eat, cook or garden together are not always easy to find or are expensive. Participants also noted that policies to restrict unhealthy food marketing on social media are currently absent.

### Leverage points

Five leverage points were identified as most important for achieving systems change by the citizens and six leverage points were identified by the professionals based on their separate CLDs developed (Table 2). Of the leverage points identified, four points showed (strong) similarities (i.e. lack of money, mental health/stress, the food environment and food skills).

**Table 2:** Leverage points identified as most important by citizens and professionals

Subtheme	Dynamic	Leverage points: Citizens	Leverage points: Professionals
Resources and daily living conditions	Scarcity and wellbeing	Lack of money	Lack of money/debts
Mental and physical wellbeing	Scarcity and wellbeing	Mental health and mental care	Mental health and stress
Social and societal factors	Scarcity and wellbeing		Understanding how scarcity affects people
Mental and physical wellbeing Social and societal factors	Scarcity and wellbeing Social and societal environment	Mental health and social support	
Food environment	Food environment	Food environment (e.g. marketing)	Exposure unhealthy food/marketing (legislation)
Individual behaviour and characteristics	Social and societal environment	Education/information	Food-skills
Social and societal factors	Social and societal environment		Social-cultural norms

Study: Why healthy eating is not equally attainable for all: A participatory systems perspective on dietary behaviour of people living on a low income in the Netherlands, and recommendations for systemic change, Ede the Netherlands, 2024–2025

### Proposed actions to change the system

Based on the CLDs and the identified leverage points within them, a total of 30 actions were formulated by the participants. All actions related to one of the three key dynamics as identified by the researchers. The descriptions of all actions, along with the key dynamics they address, are presented in Table 3.

**Table 3:** Actions proposed by citizens and professionals, categorised according to the dynamics ‘scarcity and wellbeing’, ‘food environment’, and ‘social and societal environment’

**Actions Dynamic 1- Scarcity and wellbeing**		
**It is important that:**	**Level of Action Scales model**	**Action proposed by:**
1. Health insurers reimburse mental health treatments, so that they become more accessible to everyone	Structures *Changes financial and organisational structures, which influences accessibility*	Citizens
2. The municipality and care organisations* pay more attention to the prevention of (serious) health problems within mental health care	Goals *Reflects a shift in ambition from treatment to prevention*	Citizens
3. Schools and employers (partly through government incentives) pay attention to mental wellbeing, for example through teaching programs or training	Goals *Reflects a core intention/goal to prioritise mental wellbeing*	Citizens
4. Health insurers reimburse more available hours for maternity care, with attention to and education about healthy nutrition for the baby and the family	Structures *Adjusts care provision and financial structures to include education*	Citizens
5. Health insurers include guidance by a dietician in the basic care package (also without a medical purpose), without financial thresholds	Structures *Changes the system’s accessibility and financial barriers*	Citizens
6. The national government allows the incomes and benefits of citizens to increase in line with inflation and increased fixed costs	Structures *Alters economic structures that influence resource availability*	Citizens
7. Governments* provide accessible and easy-to-find information about (applying for) allowances for citizens (for example in multiple languages ​​and formulated in simple terms)	Structures *Improves information flows and accessibility*	Citizens
8. Governments and social service organisations involve experts by experience in the development of policy and implementation of support services, in order to better align the content and language use with the target group	Structures/Beliefs *Reflects a normative shift toward valuing lived experience and co-creation, while embedding participatory practices and expert-by-experience roles into policy-making and service structures*	Citizens and Professionals
9. Social service organisations pay more attention to the personal situation of citizens, and create space for citizens to play an active management role in the support process	Structures/Beliefs *Indicates a change in attitudes toward citizen empowerment and trust, while embedding participatory practices in support structures*	Professionals
10. Governments and social service organisations work together more around an individual, with clear agreements and implementation for sharing client data between professionals, in order to guarantee integrated support services	Structures *Creates integrated service structures and formalised collaboration*	Professionals
11. Governments develop simple and accessible regulations for care and support services to citizens, with a system based on trust (such as the minimum policy in Wageningen)	Structures/Beliefs *Embeds a value of trust in governance, while simplifying structural rules*	Professionals
12. Professionals participate in training courses on the effects of scarcity on people, in order to gain more knowledge and understanding of this	Structures/Beliefs *Targets understanding among system actors and develops a training system for professionals*	Professionals
**Actions Dynamic 2- Food environment**		
**It is important that:**	**Level of Action Scales model**	**Action proposed by:**
13. Governments and employers stimulate/realize a healthy and affordable food provision in the work environment (e.g. free fruit and healthy food provision in the canteen)	Structures *Changes the physical and organisational environment*	Citizens and professionals
14. Governments and health organisations provide (financial) support to locations and organisations where children and young people come (e.g. schools, community centers, sports clubs) in realizing a healthy and affordable food environment. For example, by offering free healthy school meals at schools	Structures *Alters resource flows and organisational practices (financial support, free meals), shaping the food environment*	Citizens and professionals
15. The national government implements legislation that gives municipalities the opportunity to ban unhealthy food providers*.	Structures *Introduces regulatory mechanisms that change the availability of unhealthy food providers*	Professionals
16. The municipality makes agreements with local supermarkets and other food providers (and franchisees) about a healthier food provision, promotions and a healthier store layout	Structures *Creates new patterns and relationships between actors, influencing store environments and promotions*	Citizens and professionals
17. The national government implements legislation and regulations or subsidies or makes targeted (SMART) agreements for a healthier food provision, promotions and a healthier store layout in supermarkets and other food providers	Structures *Structural intervention through policy, regulation, and financial incentives influencing store environments*	Citizens and professionals
18. The national government implements legislation and regulations for clear and honest labelling and claims about the health (effects) of products by supermarkets and food manufacturers, with monitoring of compliance	Structures *Structural intervention through policy, regulation influencing food labelling*	Citizens
19. The municipality and local organisations make agreements with supermarkets to offer affordable and healthy meal suggestions, for example by placing ingredients together with accompanying recipe cards	Structures *Creates new relationships and information flows to stimulate healthy eating*	Citizens
20. The national government implements legislation and regulations to ban advertising for unhealthy food and to stimulate advertising for healthy food	Structures *Structural intervention through policy, regulation*	Citizens
21. The national government implements legislation that ensures that the prices of healthy food are lower than those of unhealthy food (e.g. by reducing VAT on fruit and vegetables and introducing a sugar tax)	Structures *Structural intervention through policy, regulation, and financial incentives*	Citizens and professionals
22. The national government and municipalities make agreements with supermarkets and other food providers to offer (packages of) healthy food for free or at a low price that would otherwise be thrown away	Structures *Creates new patterns and relationships between actors, that reduce waste and increase availability/affordability of healthy foods*	Citizens
**Actions Dynamic 3 – Social and societal environment**		
**It is important that:**	**Level of Action Scales model**	**Action proposed by:**
23. The municipality and local organisations* organise free social activities for vulnerable groups, with opportunities to talk to others and make social contacts	Structures *Creates new social opportunities and interaction patterns, reducing isolation and strengthening networks*	Citizens
24. The municipality and local organisations organise accessible initiatives in the field of affordable and healthy eating and/or cooking together (e.g. healthy cooking and eating evenings in community centers)	Structures *Changes the local environment and social practices, enabling shared healthy eating experiences*	Citizens and professionals
25. The municipality maps out existing local initiatives (regarding social support/meeting and healthy eating/cooking), with an analysis of possible collaborations or additional initiatives	Structures *Improves coordination and information flows between actors, shaping systemic connectivity*	Citizens
26. (Primary) schools pay structural attention to healthy food, including a healthy food offering and education programs on healthy eating and cooking	Structures *Institutionalizes healthy food practices and education, embedding them in school systems*	Citizens and professionals
27. The municipality includes neighborhood-specific information in the local (neighborhood) newspaper, e.g. about healthy eating, opportunities to use a vegetable garden, money management and applying for allowances	Events *Addresses individual behaviour of people to eat more healthily*	Citizens
28. Governments provide information about healthy eating/living on a low budget via national and regional media, from a reliable source	Events *Addresses individual behaviour of people to eat more healthily*	Citizens
29. The municipality uses a community approach to stimulate healthy eating in the neighborhood, with attention to social and cultural eating habits, norms and values ​​of different communities	Structures/Beliefs *Reflects a normative shift, valuing social and cultural norms and community engagement, while institutionalizing community-based partnerships and processes*	Professionals
30. The municipality and local (food) organisations organise an accessible course for citizens on food skills, such as healthy cooking on a limited budget	Structures/Events *Creates new learning opportunities and information flows within the system*	Professionals

Study: Why healthy eating is not equally attainable for all: *A participatory systems perspective on dietary behaviour of people living on a low income in the Netherlands, and recommendations for systemic change, Ede the Netherlands, 2024–2025*

*Aid organisations are organisations that provide support to people who need help, for example in the areas of health, welfare, social issues or societal problems

*Governments include both the national government and the municipality

*Municipalities and health organisations can play a role in lobbying for any actions aimed at the national government

*Local organisations refer to organisations that are active at the municipal level and focus on the specific needs and challenges of a local community, e.g. neighborhood and community centers, food banks, food and social organisation

*Events relate to the observable issues (behaviours and proxy outcomes) and are symptoms of the system. Structures relate to the patterns, relationships, information flows and physical structures that cause events to occur. Goals refer to the ambitions or targets that the system (or parts within the system) are working towards. Beliefs, norms, values and attitudes of systems architects (i.e. those who influence the structure and workings of a system) that cause the system to function as it does

Participants proposed 12 actions *(*40% of total actions proposed*)* related to the dynamic scarcity and wellbeing. These actions focus on areas such as the prevention of physical and mental problems, improving accessibility of care, and strengthening cooperation between professionals and experts by experience. Most of these actions (7) were proposed by citizens, for instance related to the prevention of and care for physical and mental problems, maternal/family care, and providing financial security. Professionals suggested four actions, related to improving care and support services and increasing the understanding among professionals about the impact of living in scarcity. The action to involve experts by experience in the development of policy and implementation of support services was recommended by citizens as well as professionals.

Participants formulated *10* actions (33% of total actions proposed) to create a healthier food environment, so that people are less exposed to and susceptible to unhealthy food choices. Citizens as well as professionals proposed five common actions to create a healthier food environment, related to healthy food provision, promotion and prices. In addition, citizens specifically suggested actions related to food labelling and claims, advertising, and agreements with supermarkets (e.g. about offering affordable foods) whereas the professionals proposed an action about national legislation that gives municipalities the opportunity to ban unhealthy food providers.

*Eight* actions (27% of total actions proposed) were proposed to improve the social and societal environment, which help to build social connections, develop food skills, and increasing interest and motivation for healthy eating. Professionals as well as citizens proposed two common actions about healthy food provision in schools and organising accessible cooking/eating initiatives. In addition, citizens recommended four actions (related to providing information about healthy eating/living on a low income, social activities and mapping out existing social and food initiatives) and professionals formulated two actions (i.e. a community approach and a food skills course).

Of all the actions proposed, most actions (67% ;20 actions) correspond to the ‘structures’ level of the Action Scales Model, because they relate to the patterns, relationships, information flows and physical structures (e.g. food environment) that influence dietary behaviour. Five actions correspond to the ‘structures’ as well as ‘beliefs’ level (changing attitudes, values of system architects), two actions relate to the ‘goals’ level (reflecting shifts in ambitions or targets), two actions to the ‘events’ level (because they address individual behaviour of people) and one action to the ‘events’ as well as ‘structures’ level [44].^2^ Both citizens and professionals formulated actions at the ‘beliefs’ and ‘structures’ level of the Action Scales Model [44]. The two actions corresponding to the ‘events’ level (in the social and societal environment) and the two actions corresponding to the ‘goals’ level (in the scarcity and wellbeing dynamic) were proposed by citizens. The action corresponding to the ‘events’ as well as ‘structures’ level (in the social and societal environment) was proposed by professionals.

## Discussion

This study gained insights into the complex system dynamics underlying dietary behaviour of people on a low income, from the perspective of both citizens with experiential knowledge and professionals. These perspectives overlapped and complemented each other well, leading to an integrated CLD that provides in-depth insights into the three key dynamics that explain why the current system complicates healthy eating for people on a low income. These dynamics included: (1) scarcity and wellbeing, (2) the food environment and (3) the social and societal environment. Moreover, the perspectives from citizens and professionals showed which actions are needed to facilitate healthy dietary behaviour among people living on a low income.

As far as we are aware, this is the first study specifically addressing the system underlying dietary behaviour of people on a low income, from the perspectives of citizens with experiential knowledge and professionals in the field. Nevertheless, some of the subthemes or dynamics identified are in line with or similar to other system-based studies among lower socioeconomic groups [16, 17, 22]. For instance, experiencing scarcity and the influence of that on (mental) health and wellbeing (e.g., stress), the influence of the social environment and food environment (e.g., food accessibility, availability, marketing and prices) appeared as important dynamics in other studies as well [16, 17, 22]. However, in addition to commonly addressed food environment aspects, our study specifically showed the role of misleading labels/claims on food packages and uncertain/contradictory information about healthy/unhealthy foods, which could cause stress among people who have to make ends meet on a low income. Other literature has indeed shown that exposure to misleading information can lead to feelings of confusion and stress, affecting individuals' mental wellbeing [45]. Moreover, our study addresses the role of social media to unclear/contradictory information about (un)healthiness of foods. Indeed, studies show that food and nutrition are popular topics on social media creating challenges related to misleading information [46], and with that contribute to the formation of misconceptions, but also to negative emotional associations (e.g. fear, anger, panic) [47, 48].

Our study builds on the literature by providing systemic insights of how mental health influences dietary behaviour, integrating findings that were previously reported in isolation. For example, highlighting specific and multiple dietary behaviours as a result of experiencing financial strain, worse mental health or stress (e.g., skipping meals and hunger, unhealthy eating as coping mechanisms, unhealthy eating to compensate/mask), mechanisms which can also be found in other literature [17, 49–52]. In addition, our study also illustrates the subsequent effect of mental health and stress on headspace and capacity to act thereby impacting dietary behaviour. Indeed, financial strain can lead to chronic stress, which can lead to losing the capacity to give long term goals such as optimal health, full consideration [16, 53–55]. This may make individuals in lower socioeconomic economic groups especially vulnerable to unhealthy food environments [2, 56, 57], which are sustained by the food industry's prioritisation of the production and sale of unhealthy foods and by a lack of legislation and regulation in this area, as shown in our study and in other literature [58–60].

Furthermore, what our study specifically highlights is the significant influence of a lack of societal and professional understanding of the effects of scarcity, contributing to policies and support services that often do not adequately meet the needs of people living on a low income and to prejudices about poverty in society and among professionals. Indeed, other studies also addressed the issue of policies not aligning with the preferences and needs of people in disadvantaged socioeconomic positions, which creates a gap between communities and government institutions, and increased institutional distrust and distance between social groups [16]. Current policies and support services in the Netherlands, resulting from a prominent neoliberal political ideology, reflect the lack of understanding the effects of scarcity, as policies to reduce health inequalities require high levels of self-reliance of citizens, based on the belief that increasing knowledge or providing more information will lead to changes in individual behaviour and lifestyle [61–63]. However, these policies do not take into account the insecure socioeconomic contexts people live in affecting the mental burden people can handle, nor the high amount of choices, unhealthy temptations and expected actions people are confronted with, influencing people’s capacity to act [63, 64]. The lack of understanding of what it is like to live in socioeconomic disadvantaged conditions and as a result misaligned policies, is likely caused by an underrepresentation of disadvantaged populations in positions of power and policy-making processes [16, 64–68]. It is therefore of utmost importance to incorporate lived experiences of people in disadvantaged socioeconomic conditions in research and during policy-making processes to ensure their needs and preferences are considered and policies are developed that address the social determinants of health (e.g., improved access to suitable housing) [64, 65].

### Strengths and limitations

An important strength of this study is that it comprehensively illustrates the complexity of the system facilitating unhealthy dietary behaviour among people on a low income, by showing the interrelatedness between living in scarcity, reduced physical and mental wellbeing, an unhealthy food environment, and social and societal barriers. This comprehensive and holistic approach allows for a richer understanding of the structural and contextual factors and dynamics influencing dietary behaviour among people with low income, strengthening the study’s relevance for both research and policy development.

Another important strength is that it gained insights into this system and its dynamics through the perspectives and lived experiences of citizens and professionals, whereas other related studies used literature or independent expert views to gain insights into underlying health-related systems among lower socioeconomic groups [16, 17, 22]. This has led to additional detailed in-depth insights and concrete recommendations for systemic change, which could not have been obtained otherwise.

Moreover, a strength of this study is that we specifically invited citizens with experience of making ends meet on a low income, considering the limited research among low income groups. At the same time, future research could benefit from involving other professionals (e.g., general practitioners) and a more diverse group of citizens facing different forms of socioeconomic disadvantage, as this may provide additional insights into the underlying system and actions needed for systemic change.

In addition to these strengths, our study also has some limitations. First, our study took place in a specific context, namely the Veldhuizen neighbourhood in the municipality of Ede. While some factors may be context specific, it is expected that many of the identified dynamics would also emerge in similar low-income neighbourhoods in other municipalities, as they align with structural and systemic issues described in previous studies. Future research in other municipalities and/or countries may provide additional insights and confirm or nuance these findings, and as such strengthening the evidence base.

Second, a selected group of professionals and citizens attended our sessions. Some professionals which we intended to recruit, declined our invitations (e.g., due to a lack of time) or did not respond to our invites. Moreover, the characteristics of the participating citizens showed limited diversity in age (mostly older adults), gender (predominantly female) and ethnicity (mainly Dutch).

Third, we had a limited amount of time during the GMB sessions and could only organise three GMB sessions in total, which could have had implications for the ability of participants to fully understand the concept of system thinking. This might have had impact on the system levels addressed by the actions, as in line with other studies, most actions were formulated at the ‘structures’ level (seventeen), indicating the tendency of people to address lower hanging fruit actions that are often easier to implement [24, 25]. However, this fixed and limited amount of time could also have made it feasible for the professionals and citizens to participate [24].

Fourth, participants self-identified as experiencing or having experienced difficulties with making ends meet, and talking about making ends meet during the GMB sessions can have induced feelings of shame or discomfort. To minimise these potential risks, during the sessions the focus was on dietary behaviour (and not socioeconomic insecurity) and the factors that may influence that behaviour, which may be related to socioeconomic insecurity. We asked participants in general about which factors they think that have an influence on dietary behaviour, and did not specifically ask participants to bring forward their own personal experiences with making ends meet. (Professionals were asked similar questions as the citizens). Furthermore, to foster a safe and comfortable atmosphere, we organised the sessions at the familiar and accessible community centre in Veldhuizen, with time for informal introduction and shared meal prior to the sessions, and we explicitly emphasised that participants were free to decide what they wished to share and were under no obligation to disclose anything they did not feel comfortable with.

Fifth, the participatory nature of the study required careful attention to representation and power dynamics. To ensure that the voices of citizens on a low income were meaningfully incorporated, we used several design and facilitation strategies. To reduce practical barriers for attending the sessions, sessions were held in familiar and easily accessible community locations with compensation for participation and the availability of a warm meal. Moreover, GMB sessions with citizens and professionals were conducted separately, allowing citizens to speak freely about their lived experiences without perceived judgment or institutional presence. During data analysis, the researchers explicitly considered which factors, subthemes and actions emerged from citizen and professional perspectives, and reported on these (e.g., see Appendix 3) to ensure that professional interpretations did not overshadow citizens’ perspectives. At the same time, conducting separate sessions also present a limitation, as there was limited opportunity for joint discussion (except at the end of the final GMB session) and co-creation of solutions between citizens and professionals. More extensive integrated dialogue sessions would be valuable in future research.

Finally, our study may have raised expectations because of engaging citizens in a project that explores system-level changes and did not include a follow-up trajectory to investigate the action implementation process [25]. To prevent this, we explicitly communicated throughout the project that the study’s scope is exploratory and that we aimed to gain first insights into the system and potential actions, rather than implement these actions within the project period. Furthermore, we included all proposed actions in a Dutch non-scientific report [69] and made these results available to the municpality of Ede and participating organisations. The municipality of Ede expressed interest in using the findings to inform their ongoing work on positive health and the food environment and organised at their initiative a final end meeting in which we presented all study results to citizens and professionals together, allowing for reflection and discussion how to take the actions further.

In future research, long-term process and effect evaluations of action implementation are needed, which monitor changes in the system, and take into account both expected and unexpected effects of policies and interventions [24]. Furthermore, future studies could deepen our understanding of the link between our study findings with the local context, for example by incorporating characteristics of the food environment and social support system in Ede.

### Implications for policy and practice

Integrating lived and professional experiences in our study led to the recommendation of 30 actions for systemic change, substantiating evidence that actions at different system levels (i.e., beliefs, goals, structures and events) are needed to ensure greater impact, as there is no single solution to break through the current system [16]. System change towards healthier dietary behaviour among low income groups requires coordinated, mutually reinforcing actions across system levels, as well as alignment of food environment policies with policies tackling scarcity, wellbeing and societal/social determinants [70]. Such alignment and coordination can collectively create a tipping point where beliefs and goals evolve in favour of facilitating healthy dietary behaviour among people who have to make ends meet on a low income [71].

Furthermore, the recommended actions in our study substantiate that it is important that system change takes place at both national level (e.g. legislation and regulations) and local level (e.g. neighbourhood initiatives). Different actors can contribute to the implementation of the proposed actions, such as the national and local government, health care insurers, support and care services, the food industry, food suppliers, and civil society organisations. This thus requires an integrated plan in which dynamics such as the food environment, scarcity and well-being, and the social and societal environment are approached in a coherent manner and a collective, coordinated and intersectoral effort to achieve sustainable and impactful results. In this, it is essential to involve professionals as well as citizens with experiential knowledge in the development of policy and support services in order to increase mutual understanding and to ensure that the actions are well aligned with lived realities.

## Conclusion

Based on the perspectives of citizens and professionals, this study shows that the dynamics of living in scarcity and reduced wellbeing, an unhealthy food environment, and social and societal barriers create a complex system that sustains and reinforces unhealthy eating behaviours among people who have to make ends meet on a low income. Citizens with experiential knowledge and professionals proposed 30 actions addressing these dynamics for systemic change, requiring a collective, coordinated and intersectoral approach to achieve sustainable and impactful results. In this, it is essential to involve professionals as well as citizens and/or experts by experience in the development of policy and support services in order to increase mutual understanding and to ensure that the actions are well aligned with lived realities. Future research is needed to gain further insights into the system and substantiate the evidence-base by including more perspectives and applying similar systems-based studies in other contexts. It would also be valuable to evaluate real-life policy processes and action implementation, to generate more evidence on the effectiveness of systems-based participatory approaches.

## Supplementary Information

Additional file 1.

Additional file 2.

Additional file 3.

## Data Availability

Data supporting the conclusions of this article are largely provided in the manuscript and its supplementary materials. Additional anonymised data, not included therein, are available from the corresponding author upon reasonable request.

## Abbreviations

CLD: Causal Loop Diagram
GMB: Group Model Building
STICKE: Systems Thinking In Community Knowledge Exchange

## Footnotes

1 In the Netherlands, the 120% threshold is often used to define low-income groups, to determine eligibility for social or municipal support programmes, and in local poverty policies. This is because people who earn just above the minimum wage often still struggle to make ends meet. (https://onderzoek.amsterdam.nl/publicatie/armoedemonitor-2021).

2 This adds up to 31 actions, as one action is related to both the beliefs and structures levels.
