# Supplementary material for: Why healthy eating is not equally attainable for all: a participatory systems perspective on dietary behaviour of people living on a low income in the Netherlands, and recommendations for systemic change
Source: Arch Public Health. 2026 Jan 28;84:46. doi: 10.1186/s13690-026-01838-x (PMC13543430; doi:10.1186/s13690-026-01838-x)
Supplement: Supplementary file 1 [file 13690_2026_1838_MOESM1_ESM.docx]

## **Additional file 1: General Protocol GMB sessions**

**Session 1a -** Systemic factors underlying healthy dietary behavior of people living in socioeconomic insecurity, from the perspective of professionals in city/municipality [X]

| **Central research question** | **Which factors influence healthy dietary behavior of people living in socioeconomic insecurity in municipality [X]?** |
| --- | --- |
| **Aims session 1** | Identification of systemic factors underlying healthy dietary behavior of people living in socioeconomic insecurity from the perspective of professionals in city/municipality [X]  Creating a systems map together |
| **Input session 1** | Experiences and perspectives of professionals in city/municipality [X] |
| **Output session 1** | Variable list with the most important factors that have an influence on healthy dietary behavior of people living in socioeconomic insecurity.  Systems map (Causal Loop Diagram) of professionals |

**Global programme (session in the morning or afternoon, ca. 3 hours)**

**Part 1: Plenary**

- General introduction of the session, acquaintance, explanation and aim of the session
- Explanation of the complexity of dietary behaviour and the multiple factors influencing this behaviour (e.g., our food environment, or daily living conditions); Introduction of the Nutrition Equity Framework (simplified);
- Explanation systems thinking and aim of today
- Ask participants: *What are your expectations of this and next session? What do you hope to achieve?*

**Presentation slides (SD):**

- opening - 2 min
- program of today - 2 min
- who are we/you - 4 min
- FOODPATH aim and research questions - 2 min
- Population health and unhealthy diets as risk factor – 3 min
- (un)healthy diets, dietary guidelines – 2 min
- complexity of influences on dietary behavior – 1 min
- (simplified) Nutrition Equity Framework NEF – 2 min
- NEF Socio-political context – 3 min
- NEF Social stratification (socioeconomic pos.) - 3 min
- NEF Experiences of (in)equity – 3 min
- NEF Daily living conditions and behaviour and practices – 3 min
- Introduction system thinking and video – 5 min
- Systems thinking; iceberg model – 2 min
- Solutions? (Aim of this session) - 1 min
- Example of a systems map and other results of this study – 2 min
- Expectations of this session? - 5 min

*Break*

**Part 2: Working together**

**Part 2a: identify factors**

- Introduction and explanation part 2a
- Script: [Scriptapedia/Variable Elicitation](https://en.wikibooks.org/wiki/Scriptapedia/Variable_Elicitation)
  - The central question is: **Which factors influence healthy dietary behavior of people living in socioeconomic insecurity in city/municipality [X]?**
  - *We ask you to write down all the factors you are thinking about that may influence healthy dietary behavior of people living in socioeconomic insecurity in city/municipality [X].*
  - Each participant individually makes a list of factors
  - Participants discuss the most important factors with their group and make a list of the 10 most important factors per group
  - Discuss the factors in plenary

**Part 2b: how are the factors related?**

- Introduction and explanation part 2b
- Script: [creating causal loop diagram from variable list](https://en.wikibooks.org/wiki/Scriptapedia/Creating_Causal_Loop_Diagram_from_Variable_List)
- *Now we can start working on your own factors that you just identified. For each factor we will look at how it relates to the other factors that you have also mentioned (some will be related, some will not)*
- Closure; Script: [Next steps and closing](https://en.wikibooks.org/wiki/Scriptapedia/Next_Steps_and_Closing)

**Session 1b –** Systemic factors underlying healthy dietary behavior of people living in socioeconomic insecurity, from the perspective of citizens in city/municipality [X]

| **Central research question** | **Which factors influence healthy dietary behavior of people living in socioeconomic insecurity in municipality [X]?** |
| --- | --- |
| **Aims session 1** | Identification of systemic factors underlying healthy dietary behavior of people living in socioeconomic insecurity from the perspective of citizens in city/municipality [X]  Creating a systems map together |
| **Input session 1** | Experiences and perspectives of citizens in city/municipality [X] |
| **Output session 1** | Variable list with the most important factors that have an influence on healthy dietary behavior of people living in socioeconomic insecurity.  Systems map (Causal Loop Diagram) of citizens |

**Global programme (morning or afternoon or evening session, ca. 3 hours): Similar as the programme of the session with professionals (please see above)**

**In between session 1 and session 2:**

- Researchers finalize systems map; making the CLD’s neater/clearer *(based on data from forms, notes and audio recordings)*
  - Continue to build the system map by confirming relationships between factors and identifying new factors / relationships.
  - What are the stories of these relationships?
  - Include the polarities/are polarities correct?
  - What is missing?
  - Where are the reinforcing / balancing loops?
- Researchers identify leverage points where actions can be taken to initiate systems change; Script: [Places to intervene](https://en.wikibooks.org/wiki/Scriptapedia/Places_To_Intervene) *(we have let the participants choose leverage points themselves in session 2)*

**Session 2: citizens & professionals formulate actions for system change**

| **Central research question** | **Which measures/actions are needed to make healthy dietary behavior of people living in socioeconomic insecurity in city/municipality [X] easier?** |
| --- | --- |
| **Aims session 2** | **Citizens and professionals in city/municipality [X] formulate system-based actions to make healthy dietary behavior for people living in socioeconomic insecurity easier** *--> focus on system change as a result of the GMB* |
| **Input session 2** | CLD’s of session 1, including feedback loops; |
| **Output session 2** | - Final CLD’s, agreed upon by the groups of people - Leverage points identified by participants - Participants formulate for leverage points system-based actions to make healthy dietary behavior for people living in socioeconomic insecurity easier --> action list addresses different levels of the system - Reflection of the two groups on each other’s CLD’s and actions formulated |

**Global programme (morning or afternoon session, ca. 3 hours)**

**Plenary:**

- General opening of session with explanation and aim of the session

**Part 1: Separately for citizens and professionals**

- 30 min. Presenting and discussing CLD and loops – what is missing, what should be adjusted?
- Discussing most important leverage points – individually and plenary
- script: [transferring group ownership from one image to another](https://en.wikibooks.org/wiki/Scriptapedia/Transferring_Group_Ownership_from_One_Image_to_Another)
- Formulating actions; Comparable script: [Actions ideas](https://en.wikibooks.org/wiki/Scriptapedia/Action_Ideas)
  - 5 minutes: explanation
  - 10 minutes: formulating actions individually
  - 20 minutes: discussing actions in groups
  - 25 minutes: presenting/discussing actions

*This part can be organized in one room but divided into separate groups of citizens and professionals or in two separate rooms.*

**Part 2: Jointly with citizens and professionals**

- 45 minutes: Reflecting on the CLD’s and actions of each other: differences and similarities
- *How to continue? What is needed to implement the actions? How to continue within city/municipality [X]? What do you need?*
- Closure; Script: [Next steps and closing](https://en.wikibooks.org/wiki/Scriptapedia/Next_Steps_and_Closing)
