# Supplementary material for: Why healthy eating is not equally attainable for all: a participatory systems perspective on dietary behaviour of people living on a low income in the Netherlands, and recommendations for systemic change
Source: Arch Public Health. 2026 Jan 28;84:46. doi: 10.1186/s13690-026-01838-x (PMC13543430; doi:10.1186/s13690-026-01838-x)
Supplement: Supplementary file 2 [file 13690_2026_1838_MOESM2_ESM.docx]

## **Additional file 2: Subthemes and observed similarities and differences between citizens and professionals**

Subtheme 1: Government and policy

Six factors were identified within the government and policy subtheme, of which none were proposed by both groups. Three factors were related to food policies, of which *professionals* named **local food policy** (of municipalities/regions) and **legislation/national food policy**, whereas *citizens* mentioned **lobbying by health organisations** that contribute to the implementation of these policies. In addition to food policies, *professionals* mentioned the factors **social policy and regulations** and **organisation and accessibility (e.g. affordability and findability) of care,** and *citizens* identified the factor (the accessibility of) **low-threshold medical care**, which are essential for preventing physical and mental health problems (subtheme 6), especially for people who are financially vulnerable.

Subtheme 2: Food industry

This subtheme consists of two factors related to the influence of the food industry which were brought forward by *citizens as well as professionals*. *Both groups* mentioned **the power of the food industry** which refers to its major role in shaping the food environment and the influence it exerts on national legislation and food policy. Moreover, participants indicated that the **food industry and food suppliers often prioritize the production and sale of unhealthy food,** as profit is often considered more important than the well-being of consumers, which can result in an unhealthy food environment (see subtheme 4).

Subtheme 3: Social and societal factors

This subtheme includes thirteen factors of which six factors were mentioned by citizens as well as professionals. *Both groups* mentioned that social factors such as the degree of **health of the** **eating behaviour of the social network, the healthiness of cultural eating habits, upbringing about (un)healthy eating, and education at schools** about healthy eating, play an important role in eating behaviour, with *professionals* specifically mentioning **wanting to join in unhealthy eating**. In addition, *both groups* indicated the influence of factors like **having a social network and experiencing social support,** with *professionals* specifically indication **the influence of participating in society,** and *citizens* the role of **eating, cooking or gardening together**. In addition, participants also identified the influence of societal factors, including the increasingly **individualistic society** (*professionals)* and as a result of that the increased **use of social media** (*both groups*)**,** leading to higher exposure to information about and marketing for unhealthy food. Furthermore, participants indicated that factors including **prejudices about poverty** *(citizens)*, u**nderstanding the impact of living in scarcity** *(professionals)* **and involving vulnerable groups (as experts by experience) in the development of policy** *(professionals)* can impact some’s mental wellbeing (theme 6) and are essential for accessibility of care and support services (subtheme 1).

Subtheme 4 – Food environment

The subtheme food environment includes eleven factors of which six factors were brought forward by both groups. *Citizens as well as professionals* mentioned factors maintaining the predominantly unhealthy food environment, including the low level of **health of food providers in an environment, (un)healthy food availability within these providers**, and **dependency on the food bank's supply**. In addition, *professionals* specifically mentioned the low **accessibility of healthy food providers compared to unhealthy food providers**. Moreover, *both groups* indicated that the **price of healthy food is high compared to unhealthy food**, and that there is a lot of **marketing for unhealthy food.** *Citizens* specifically indicated the influence of **misleading labels/claims on packaging** which provide **unclear or contradictory information** about how healthy a product actually is. In addition, **accessible neighborhood initiatives,** such as **communal cooking or eating moments and accessible local vegetable gardens** were mentioned by *professionals* to contribute to more contact with healthy food. The largely unhealthy food environment leads, according to *both groups*, to high **individual exposure to unhealthy foods.**

Subtheme 5-Resources and daily living conditions

Of the nine factors in this subtheme, eight factors were proposed by *both groups*. Citizens and professionals mentioned factors related to resources such as a **lack of money** and **less (relatively) budget for healthy food,** with only *professionals* specifically mentioning **financial debts**. In addition, *both groups* mentioned the influence of living in **unfavorable housing conditions**, living in a **neighborhood with a low SES** (socioeconomic status), the amount (lack) of **free time** that someone has, **working long hours** to make ends meet, and little possibilities for someone to undertake **healthy leisure activities**.

Subtheme 6- Mental and physical wellbeing

This subtheme contains nine factors that relate to how someone feels mentally and physically, of which six factors were mentioned by *citizens as well as professionals*. When it comes to physical well-being, poorer **physical health**, and reduced **mobility** were brought forward by *both groups*. However, *citizens* additionally mentioned **hunger** as an important factor. Mental well-being includes factors like poor **mental health** and **stress (***citizens and professionals).* Both mental health and stress influence someone's **headspace** *(professionals)* **and ability to act (***citizens and professionals)* (e.g. making healthy food choices)**.** In addition, *both groups* indicated that **shame** can contribute to poorer mental health and *citizens* indicated that this can cause someone to not **dare to ask for (**practical, emotional or social ) **support.**

Subtheme 7: Individual characteristics and behaviour

This subtheme consists of twelve factors, of which nine factors were indicated by *both groups*. First of all, *citizens as well as professionals* indicated that **basic skills** and **food skills and knowledge about healthy eating**, are important, the latter also to ensure that someone becomes more **interested in and motivated for healthy eating** and therefore gives more **priority to healthy eating**. Furthermore, *both groups* mentioned the influence of factors as **focusing on foods that promote satiety instead of on its healthiness (as a result of hunger), and tending towards convenience foods** (as a result of limited space and opportunities to prepare healthy meals), with *citizens* specifically indicating the influence of having **to skip meals** due to a lack of money. In addition, *both groups* mentioned the behavioural factor developing **unhealthy addictions** (e.g. smoking or alcohol) as a response to stress, and *citizens* also mentioned **unhealthy eating** as **coping mechanism**, and **eating unhealthy as a way of compensating or to mask** the fact to be uncapable of keeping up with things. *Citizens and professionals* indicated that these factors can make people **susceptible to making (impulsive) unhealthy food choices**, which can maintain or increase the **preference for fat/sugar/salt in food.**
