# Supplementary material for: Why healthy eating is not equally attainable for all: a participatory systems perspective on dietary behaviour of people living on a low income in the Netherlands, and recommendations for systemic change
Source: Arch Public Health. 2026 Jan 28;84:46. doi: 10.1186/s13690-026-01838-x (PMC13543430; doi:10.1186/s13690-026-01838-x)
Supplement: Supplementary file 3 [file 13690_2026_1838_MOESM3_ESM.docx]

## **Additional file 3: Table with factors**

| Nr. | Factor | Definition  + *Explanation/relevance* | Professionals and/or citizens | Influence on | Influenced by |
| --- | --- | --- | --- | --- | --- |
| Theme 1: Government and policy | | | | | |
| 1 | Legislation/  national food policy | National government laws, regulations, and strategies that ensure food supply, safety, and quality, and promote the health of the population. For example, by taxing unhealthy products, restricting the marketing of unhealthy foods, or ensuring that municipalities can ban unhealthy food providers from establishing themselves. | Professionals | Marketing unhealthy food (-)  Availability of healthy food in suppliers (+)  Health of food suppliers in the area (+)  Price healthy vs. unhealthy food (-)  Misleading labels/claims on packaging (-)  Power of the food industry (-)  Local food policy (+)  Education of schools on healthy eating (+) | Lobby health organizations (+)  Power food industry (-) |
| 2 | Local food policy | Measures and strategies by local governments to promote the availability, accessibility and consumption of healthy food within a specific region. This includes, for example, providing vegetable gardens and regulating the establishment of healthy versus unhealthy food providers. | Professionals | Health food providers in the area (+)  Accessibility of neighborhood initiatives cooking/eating (+)  Accessibility of local vegetable gardens (+)  Availability of healthy food in providers (+)  Education schools healthy eating (+) | Legislation/national food policy (+)  Lobby health organizations (+) |
| 3 | Lobby health organizations | Health organization lobbying refers to the activities of these organizations to influence policymakers and legislators to develop and implement health-related legislation and regulations.  For example, health organizations can lobby for legislation and policies that contribute to an environment with a healthier food supply. | Citizens | Local food policy (+)  Legislation/national food policy (+) | NA |
| 4 | Social policy/  legislation | Social policy refers to the set of measures and laws by the government or other social organizations that ensure that people can meet their basic needs, such as income and housing. It aims to reduce inequality and support vulnerable groups and individuals. | Professionals | (Accesibility) care and support services (+) | Involvement of vulnerable target group in policy (+) |
| 5 | (Accesibility) care and support services | The degree of accessibility (e.g. affordability and findability) of support and care for problems such as debts or social difficulties. Assistance (e.g. debt assistance or social work) is often provided and arranged by local (government) organizations. The organisation and accessibility of assistance is influenced by regulations (e.g. Wmo).  *Accessible assistance can relieve stress and prevent worse problems from arising.* | Professionals | Mental health (+)  Dare to ask for support (+) | Basic skills (+)  Social policy/regulations (+)  Dare to ask for support (+)  Understanding what scarcity does to someone (+)  Involvement of vulnerable target group in policy (+) |
| 6 | (Accessibility) low-threshold medical care | The degree of ease with which people can obtain basic medical care without barriers (such as costs or complicated procedures). In the context of prevention of more serious mental/physical problems, it is precisely important that low-threshold medical care is accessible to everyone. Health insurance schemes (e.g. paying the deductible) play a role in this. | Citizens | Mental health (+)  Physical health (+) | Lack of money (-)  Basic skills (+) |
| Theme 2: Food industry | | | | | |
| 7 | Power food industry | The major influence the food industry has on food consumption, through food production, prices, marketing, and supply, to policy influence. | Professionals and citizens | Priority unhealthy food industry/providers (+) | Legislation/national food policy (-) |
| 8 | Priority unhealthy food industry/providers | The food industry and food suppliers are often focused on making as much money as possible instead of on the well-being/health of consumers. Therefore, they focus on the production and sale of unhealthy food, in order to meet the preference that most people have for fat, sugar and salt. | Professionals and citizens | Availability of healthy food in suppliers (-)  Marketing unhealthy food (+)  Price healthy vs. unhealthy food (+)  Health food suppliers in area (-)  Misleading labels/claims on packaging (+) | Power of food industry (+)  Preference for fat/sugar/salt (+) |
| Theme 3: Social and societal factors | | | | | |
| 9 | Healthy eating behaviour social network | This refers to how healthy the eating habits of your social network are, for example how much, often and healthily your family or friends eat. The eating habits of your social network have a great influence on how you deal with healthy eating yourself, in social situations or separately.  *On the one hand, people around you can be a source of inspiration to eat healthy. On the other hand, if they often choose unhealthy food such as fast food, it can be difficult to eat healthy yourself.* | Professionals and citizens | Health cultural eating habits (+)  Education healthy eating (+)  Interest/motivation healthy eating (+)  Wanting to join in with unhealthy eating (-) | Health cultural eating habits (+)  Wanting to join in with unhealthy eating (-) |
| 10 | Health cultural eating habits | The health of traditions, values and habits around food from a certain culture. For example, norms regarding portion size and eating moments, the importance of eating together or the appreciation for fresh and unprocessed food. | Professionals and citizens | Health eating behaviour social network (+)  Education healthy eating (+)  Interest/motivation healthy eating (+)  Want to join in with unhealthy eating (-)  Focus on satiety instead of health of food (-) | Health eating behaviour social network (+)  Want to join in with unhealthy eating (-) |
| 11 | Upbringing healthy eating | Upbringing about healthy eating refers to the guidance and teaching of parents/caregivers of the child in the area of healthy eating.  *This includes, for example, the extent to which a child learns at home that the healthiness of food is important (as opposed to just the taste and how satiating food is), but also how to buy/prepare healthy food.* | Professionals and citizens | Food skills/knowledge healthy eating (+)  Focus on satiety instead of health of food (-)  Interest/motivation healthy eating (+)  Unhealthy eating as coping (-)  Preference for fat/sugar/salt (-) | Health eating behaviour social network (+)  Health cultural eating habits (+) |
| 12 | Education schools healthy eating | Education about healthy food in (primary) schools is the transfer of knowledge, skills and awareness about food and its impact on health.  *It is important to teach children about healthy food at a young age, because this can provide more knowledge, skills and motivation, also in later stages of life.* | Professionals and citizens | Food skills/knowledge healthy eating (+) | Legislation/national food policy (+)  Local food policy (+) |
| 13 | Social support/social network | The social relationships and contacts you have, such as friends and family, and the help you receive from them. For example, they can offer a listening ear or practical help.  *Regarding healthy eating, social contacts can, for example, stimulate someone to cook/eat together and to maintain healthy eating habits.* | Professionals and citizens | Participation in society (+)  Mental health (+)  Dare to ask for support (+)  Eating/cooking/gardening together (+) | Participation in society (+)  Eating/cooking/gardening together (+) |
| 14 | Participation in society | Participation in society refers to actively participating in social life, for example through (volunteer) work, education or social activities. It is about being involved in the community.  *Active participation can be difficult when there is, for example, shame, or the lack of basic skills or a social network.* | Professionals | Social support/social network (+)  Mental health (+)  Shame (-) | Eating/cooking/gardening together (+)  Social support/social network (+)  Shame (-)  Basic skills (+) |
| 15 | Eating/cooking/gardening together | Eating, cooking or gardening together with other people. For example, with your family, friends or neighbours.  *Eating together can ensure that meals are healthier, more varied or cheaper (due to cheap large packages). Moreover, working together on (healthy) food can be fun, strengthen social support and be educational in the context of food skills.* | Citizens | Food skills/knowledge healthy eating (+)  Participation in society (+)  Social support/social network (+)  Interest/motivation healthy eating (+) | Social support/social network (+)  Accessibility local vegetable gardens (+)  Accessibility neighborhood initiatives cooking/eating (+) |
| 16 | Individualistic society | A society in which personal freedom, responsibility and independence are more important than group interests. As in today's society, people should primarily take care of themselves (self-reliance) instead of the community. | Professionals | Use of social media | NA |
| 17 | Use of social media | Many people have a smartphone/laptop and use social media: platforms and technologies that allow information to be shared and communicated. This increases the individual exposure to information, but also marketing, as these media are also used by companies (and influencers).  *By using social media, someone also comes into contact with more information about and marketing for (often unhealthy) food products and eating habits*. | Professionals and citizens | Want to join in with unhealthy food (+)  Unclear/contradictory information health food (+)  Marketing unhealthy food | Individualistic society  Marketing unhealthy food |
| 18 | Prejudices of others about poverty | Prejudices about poverty are negative or wrong ideas that people have about poor people, such as that they are lazy or to blame for their own situation. This can be due to a lack of understanding of the (complex) causes and effects of poverty. | Citizens | Shame (+) | Understanding what scarcity does to someone (-) |
| 19 | Understanding what scarcity does to someone | The understanding of others (e.g. professionals) and yourself of what scarcity (a lack of resources such as money) can do to you. The integrality and complex coherence of different effects such as shame, lack of money, stress, debts, addictions and the susceptibility to impulsive/unhealthy food choices.  *The degree of understanding determines how bystanders and professionals deal with people who live in scarcity.* | Professionals | (Accesibility) care and support services (+)  Prejudices of others about poverty (-)  Shame (-) | Involvement of vulnerable target group in policy (+) |
| 20 | Involvement of vulnerable target group policy (+) | Actively involving people from a vulnerable target group (e.g. people with debts) in the development, implementation and evaluation of policy, so that it better matches their needs and situation. These people know from their experiences, often better than some professionals/policy makers, what is needed. | Professionals | Understanding what scarcity does to someone (+)  (accesibility) care and support services (+)  Social policy/legislation (+) | NA |
| 21 | Want to join in with unhealthy eating | Eating unhealthy because people around you do this too. For example, at social events, people often eat unhealthy and it can be difficult (due to peer pressure) to deviate from this. | Professionals | Susceptibility to (impulsive) unhealthy food choices (+) | Use of social media (+)  Health eating behaviour social network (-)  Health cultural eating habits (-) |
| Theme 4: Food environment | | | | | |
| 22 | Dependence on food bank offerings | A food bank is an organization that provides free food to people whose income is too low to buy enough food. Although many food banks have a policy of providing people with products for at least a few healthy meals, the food supply is dependent on surpluses from supermarkets, producers, and other donors.  *People who depend on the food bank for enough food may experience feelings of shame and have limited choices regarding (healthy) food products.* | Professionals and citizens | Shame (+)  Individual exposure to unhealthy food | Lack of money (-) |
| 23 | Price healthy vs. unhealthy food | The prices of healthy, nutritious foods compared to unhealthy foods. In many cases, unhealthy foods are relatively cheaper per serving, weight, or amount of calories.  *People who have a limited budget to buy enough food may therefore be inclined to buy more unhealthy foods.* | Professionals and citizens | (Relative) budget healthy food (-) | Legislation/national food policy (-)  Priority unhealthy food industry/providers (+) |
| 24 | Individual exposure to unhealthy food | Exposure to unhealthy food refers to the extent to which people come into contact with unhealthy food in their physical/digital environment. For example, by walking in the supermarket, watching commercials on television, or seeing advertisements on social media | Professionals and citizens | Susceptibility to (impulsive) unhealthy food choices (+) | Health food providers in environment (-)  Bereikbaarheid gezonde vs. ongezonde voedselaanbieders (-)  Beschikbaarheid gezond voedsel in aanbieders (-)  Marketing ongezond voedsel (+)  Afhankelijkheid aanbod voedselbank |
| 25 | Marketing unhealthy food | The strategic promotion of unhealthy foods to ensure more sales of these products. Marketing can be used in the physical and digital environment and examples are advertisements, attractive packaging, layout in stores (e.g. placing products at the checkout or at eye level on shelves), and advertisements in videos of influencers on social media.  *Marketing plays on (impulsive) choices and people who live in scarcity may be extra sensitive to this. Especially when promotions are made with lower prices of products.* | Professionals and citizens | Use of social media  Uncertainty/contradiction of health food information (+)  Individual exposure to unhealthy food (+) | Priority unhealthy food industry/suppliers (+)  Legislation/national food policy (-)  Use of social media |
| 26 | Health food providers in environment | The health of different food providers in the living, working and school environment. For example, are there mainly fast food shops and restaurants present, which often offer mainly unhealthy food, or are there more shops that offer fresh and healthy products?  *The presence of healthy providers, and the absence of unhealthy ones, can ensure that you are less tempted to choose unhealthy food.* | Professionals and citizens | Accessibility of healthy vs. unhealthy food providers (+)  Individual exposure to unhealthy food (-) | Legislation/national food policy (+)  Local food policy (+)  SES Neighborhood (+)  Priority unhealthy food industry/suppliers (-) |
| 27 | Availability healthy food in providers | The share of healthy foods compared to the total food supply in food suppliers in your living, working and school environment.  *In food suppliers, the supply is often largely unhealthy: for example, 80% of the supply in the supermarket is unhealthy and in some suppliers, such as petrol stations or cinemas, there are hardly any healthy options for sale*  *.* | Professionals and citizens | Individual exposure to unhealthy food (-) | Priority unhealthy food industry/suppliers (-)  Local food policy (+)  Legislation/national food policy (+)  SES neighborhood (+) |
| 28 | Misleading labels/claims on packages | Claims on products that can mislead consumers about the ingredients, health value or origin of a product, often to stimulate sales.  *Concrete examples are claims such as ‘rich in fibre’ or ‘no added sugar’, which emphasise one aspect, while the entire product does not necessarily have to be healthy. In addition, some people find the NutriScore labels unclear.* | Citizens | Uncertainty/contradiction in health food information (+) | Priority unhealthy food industry/suppliers (+)  Legislation/national food policy (+) |
| 29 | Uncertainty/contradiction in health food information | Too much and unclear/contradictory information about the health of certain food products or eating habits. This can make it difficult to understand which food is good or bad for your health.  *This can cause stress when you want or need to pay attention to healthy eating.* | Citizens | Stress (+)  Susceptibility to (impulsive) unhealthy food choices (+)  Food skills/knowledge healthy eating (-) | Marketing unhealthy food (+)  Misleading labels/claims on packaging (+)  Use social media (+)  Basic skills (-)  Food skills/knowledge healthy eating (-) |
| 30 | Accessibility of neighborhood cooking/eating initiatives | The degree of accessibility (affordability and findability) of initiatives to cook and/or eat together with local citizens.  *Sometimes initiatives fit in well with the needs of citizens, but are expensive or difficult to find, which still results in low turnout.* | Professionals | Eating/cooking/gardening together (+) | Local food policy (+) |
| 31 | Accessibility of local vegetable gardens | The degree of accessibility (affordability and findability) of local opportunities to use a (shared) vegetable garden. | Professionals | Eating/cooking/gardening together (+) | Local food policy (+) |
| 32 | Accessibility of healthy vs. unhealthy food providers | The extent to which people have physical access to healthy food providers, such as a greengrocer, compared to unhealthy food providers, such as fast food restaurants. This is about the distance to shops, but also about how easily people can reach them in terms of transport/mobility.  *Organic/healthy shops are often further away. This has an even greater impact if you are less mobile.* | Professionals | Individual exposure to unhealthy food (-) | Health food providers in environment (+)  Mobility (+) |
| Theme 5: Resources and daily living conditions | | | | | |
| 33 | Leisure time | The amount of time you have besides work and other daily obligations.  *Having little free time can make you more inclined to eat easy, fast and unhealthy food.* | Professionals and citizens | Tending toward convenience foods (-)  Healthy leisure activities (+) | Working hours (-) |
| 34 | Working hours | The number of hours someone works to earn (sufficient) money. | Professionals and citizens | Leisure time (-)  Lack of money (-) | NA |
| 35 | Lack of money | Having to survive on little money and not having enough money to meet basic needs or other expenses. It can be temporary or permanent and affects a person's possibilities and quality of life. | Professionals and citizens | Debts (+)  Dependency on food bank supply (+)  Shame (+)  (Relative) budget healthy food (-)  Skipping meals (+)  Stress (+)  Quality/size of living space (-)  (Accessibility) low-threshold medical care (-) | Debts (+)  Unhealthy addictions (e.g. smoking) (+)  Working hours (-) |
| 36 | Debts | Amounts that someone still has to repay because money was borrowed or bills were not paid on time. | Professionals | Lack of money (+)  Shame (+)  Stress (+) | Lack of money (+) |
| 37 | Quality/size of living space | The size and quality of the living space includes the number of rooms, how well the house can be kept clean and the presence of a garden/balcony, (noise) nuisance and the possibility of preparing meals.  *People with little money often have a smaller home or a home of lower quality. This can affect someone's well-being and the possibilities to prepare and store healthy food (e.g. freezing).* | Professionals and citizens | Physical health (+)  Mental health (+)  Healthy food preparation options  (+) | Lack of money (-) |
| 38 | Healthy food preparation options | The practical options a person has to prepare healthy meals. This may depend on available equipment to prepare and store healthy food, and available kitchen space.  *A person with limited mobility and/or physical health may have more difficulty using the options to prepare healthy meals.* | Professionals and citizens | Tending towards convenience foods (-) | Physical health (+)  Mobility (+)  Quality/size of living space (+) |
| 39 | Healthy leisure activities | Healthy leisure activities are activities that are undertaken during free time and that contribute positively to your physical, mental and social well-being. This includes physical activities, such as walking and team sports, but also relaxing and creative activities, such as reading and gardening. (e.g. relaxation, sports, outdoor activities, social activities).  *Engaging in healthy leisure activities can be more difficult for people who have to work a lot and have to make ends meet on a low income, because it takes time and sometimes money.* | Professionals and citizens | Stress (-)  Mental health (+)  Physical health (+) | Leisure time (+)  Lack of money (-) |
| 40 | SES neighborhood | The socioeconomic status (SES) of a neighborhood refers to the average socioeconomic position of its citizens, based on factors such as income and education level. A neighborhood with a low SES often has people with a lower average income and is often characterized by less access to employment, quality housing and (health) facilities.  *In low SES neighborhoods, the food supply is often less healthy than in high SES neighborhoods.* | Professionals and citizens | Health of food providers in the area (+)  Availability of healthy food in providers (+) | NA |
| 41 | (Relative) budget healthy food | The money someone has to spend on healthy food and the amount of healthy food someone can buy with it.  *Lower prices of healthy food and knowing how to buy/prepare affordable and healthy food (food skills) ensure that more healthy food can be bought on a limited budget.* | Professionals and citizens | Susceptibility to (impulsive) unhealthy food choices (-)  Skipping meals (-) | Price healthy vs. unhealthy food (-)  Lack of money (-)  Food skills/healthy eating knowledge (+) |
| Theme 6: Mental and physical health | | | | | |
| 42 | Mental health | Mental health refers to a person’s emotional, psychological, and social well-being. Good mental health helps them cope with challenges in everyday life. Poor mental health can lead to, or be associated with, problems such as depression, trauma, or chronic stress.  *Good mental health can increase your ability to engage in healthy eating.* | Professionals and citizens | Capacity to act (+)  Headspace (+)  Stress (-)  Physical health (+) | Physical health (+)  (Accessibility) low-threshold medical care (+)  Quality/size of living space (+)  Healthy leisure activities (+)  (accesibility) care and support services (+)  Dare to ask for support (+)  Shame (-)  Sociale support/social network (+)  Participation in society (+)  Unhealthy addictions (e.g. smoking) (-)  Stress (-) |
| 43 | Physical health | Physical health refers to the overall condition of the body and how well it functions. This includes, for example, the absence of illness and the presence of energy and physical endurance.  *A (chronically) poor physical health can make it more difficult to eat healthily, while you need sufficient (additional) nutrients.* | Professionals and citizens | Mobility (+)  Healthy food preparation options (+)  Stress (-)  Mental health (+) | Stress (-)  (Accessibility) low-threshold medical care (+)  Quality/size of living space (+)  Healthy leisure activities (+)  Mental health (+)  Hunger (-)  Unhealthy addictions (-) |
| 44 | Shame | Shame is an emotion that arises when someone cannot meet the expectations of others or themselves. Shame can arise, for example, if you cannot keep up with the standards of society, such as being financially independent.  *A feeling of shame can therefore be learned based on (unrealistic) standards and expectations of others. Feelings of shame can cause someone to withdraw and feel burdened to ask for help. Someone can actually experience less shame if there is a better understanding of what scarcity does to someone (by yourself and others).* | Professionals and citizens | Dare to ask for support (-)  Participation in society (-)  Mental health (-)  Unhealthy eating as compensation/masking (+) | Unhealthy addictions (e.g. smoking) (+)  Lack of money (+)  Debts (+)  Prejudices of others about poverty (+)  Dependence on food bank supply (+)  Participation in society (-)  Understanding what scarcity does to someone (-) |
| 45 | Stress | Stress is a physical and mental response of the body to challenges (e.g. financial problems), busy situations or high expectations. Although small amounts of stress can be motivating, long-term (chronic) stress can have negative effects on a person’s physical and mental well-being.  *When it comes to wanting to make healthy food choices, unclear information or information overload can cause stress. Stress can occur when someone is trying to make a good choice in a limited time or on a limited budget.* | Professionals and citizens | Susceptibility (impulsive) unhealthy food choices (+)  Unhealthy eating as coping (+)  Physical health (-)  Mental health (-)  Headspace (-)  Capacity to act (-)  Unhealthy addictions (+) | Uncertainty/contradiction in health food information  (+)  Capacity to act (-)  Physical health (-)  Healthy leisure activities (-)  Lack of money (+)  Mental health (-)  Debts (+) |
| 46 | Capacity to act | Capacity to act refers to the ability to actually put plans or goals (e.g. to eat healthy) into practice. Capacity to act is influenced by individual and environmental factors, including (mental) well-being.  *Regarding wanting to eat healthy, capacity to act can help to keep this up, despite temptations (e.g. through offers) to eat unhealthy.* | Professionals and citizens | Healthy eating priority (+)  Susceptibility (impulsive) unhealthy food choices (-)  Stress (-)  Healthy Eating Skills/Knowledge (+)  Tendency toward convenience foods (-) | Headspace (+)  Stress (-)  Mental health (+)  Basic skills (+) |
| 47 | Dare to ask for support | The ability and courage to ask others for practical, emotional or social help.  *It can be difficult to dare to ask for help if you experience shame, have few social contacts or if help is not accessible.* | Citizens | Mental health (+)  (accesibility) care and support services (+) | Social support/social network (+)  Shame (-)  (accesibility) care and support services (+) |
| 48 | Headspace | The time and quiet to think, without distraction or pressure, so that someone can make decisions (e.g. about healthy eating).  *Stress or other mental problems can make you feel less free in your head to think about healthy eating and act accordingly.* | Professionals | Capacity to act (+) | Stress (-)  Mental health (+) |
| 49 | Mobility | Mobility refers to the possibilities you have (yourself and in your environment) to move around. For example, do you have a car/bicycle or public transport at your disposal, and are you physically healthy enough to go somewhere.  *If someone is less mobile, the possibilities to buy and prepare healthy food are more limited and someone may tend more quickly towards convenience food.* | Professionals and citizens | Tending toward convenience foods (-)  Accessibility of healthy vs. unhealthy food providers (+)  Preparation options for healthy food (+) | Physical health (+) |
| 50 | Hunger | Hunger is a strong feeling of physical need for food. This happens when your body is short of energy and needs nutrients. | Citizens | Focus on satiety rather than healthy food (+)  Physical health (-) | Skipping meals (+) |
| Theme 7: Individual characteristics and behaviour | | | | | |
| 51 | Basic skills | Basic skills refer to skills such as reading, writing, arithmetic and digital skills. These skills help you to function in daily life.  *When it comes to nutrition and health, basic skills help you to acquire knowledge and understand information. For example, when it comes to requesting the right help or care, or reading labels on food products.* | Professionals and citizens | Uncertainty/contradiction information health food (-)  (accessibility) care and support services (+)  (Accessibility) low-threshold medical care (+)  Participation in society (+)  Food skills/knowledge healthy eating (+)  Capacity to act (+) | NA |
| 52 | Food skills/knowledge healthy eating | The (practical) knowledge and skills needed to handle food healthily and efficiently. It concerns everything that has to do with choosing, preparing, storing and eating healthy food. Examples include being aware of additives in processed products, being able to prepare a variety of healthy meals, understanding labels and planning healthy meals on a budget. | Professionals and citizens | Uncertainty/contradiction of information health food (-)  Tendency towards convenience food (-)  Susceptibility (impulsive) unhealthy food choices(-)  Interest/motivation healthy eating (+)  (Relative) budget healthy food | Uncertainty/contradiction information health food (-)  Education schools healthy eating (+)  Education healthy eating (+)  Eating/cooking/gardening together (+)  Basic skills (+)  Capacity to act (+) |
| 53 | Healthy eating priority | Prioritizing healthy eating means that someone consciously chooses healthy food as something important in daily life. Someone also actively takes this into account when making food choices.  *It can be more difficult to prioritize healthy eating if someone has little personal motivation for it, or a limited capacity to act due to stressful circumstances.* | Professionals and citizens | Susceptibility (impulsive) unhealthy food choices(-) | Interest/motivation healthy eating (+)  Capacity to act (+) |
| 54 | Tending towards convenience food | Having the preference or habit of choosing food that is quick and easy to prepare, such as ready-made meals, fast food or snacks.  *People are more likely to choose convenience if, for example, there is limited time or opportunity to prepare a healthy meal, or if they do not know how to make a healthy meal quickly/easily*. | Professionals and citizens | Susceptibility (impulsive) unhealthy food choices(+) | Food skills/knowledge healthy eating (-)  Leisure time (-)  Mobility (-)  Preparation options healthy eating (-)  Capacity to act (-) |
| 55 | Unhealthy addictions (e.g., smoking) | Unhealthy addictions refer to the repeated behaviour or use of products/substances that are harmful to physical or mental health. For example, addictions to smoking, alcohol, unhealthy food (junk food) and social media. Addictions are often difficult to control, even when someone is aware of the negative consequences. | Professionals and citizens | Shame (+)  Lack of money (+)  Physical health (-)  Mental health (-) | Stress (+) |
| 56 | Interest/motivation healthy eating | The inner desire to choose food products and meals that are good for your health. This inner interest and motivation are helpful in maintaining healthy eating behaviour*.*  *Interest in and motivation for healthy eating can be stimulated by, for example, more awareness/knowledge or by someone's cultural/social environment.* | Professionals and citizens | Susceptibility (impulsive) unhealthy food choices (-)  Healthy eating priority (+) | Food skills/knowledge healthy eating (+)  Education healthy eating (+)  Health cultural eating habits (+)  Health eating behaviour social network (+)  Eating/cooking/gardening together (+) |
| 57 | Focus on satiety instead of healthy food | Focusing on satiety and on quantity (calories) of food when making choices, rather than its nutritional value and health benefits. Energy-dense and processed products such as fast food often offer more satiation per dollar than healthy options such as fresh vegetables.  *People who have to make ends meet on a low income may have to focus more on foods that promote satiety than the healthiness of it in order to eat enough.* | Professionals and citizens | Susceptibility (impulsive) unhealthy food choices (+) | Upbringing healthy eating (+)  Hunger (+)  Health cultural eating habits (-) |
| 58 | Preference for fat/sugar/salt | The innate and learned tendency of people to find foods high in fat, sugar, and salt appealing. These preferences are biologically and evolutionarily determined, but are also influenced by social, cultural, and environmental factors.  *This contributes to the continued demand for and production of unhealthy foods.* | Professionals and citizens | Priority unhealthy food industry/suppliers (+)  Susceptibility (impulsive) unhealthy food choices(+) | Upbringing healthy eating (-)  Susceptibility (impulsive) unhealthy food choices(+) |
| 59 | Susceptibility (impulsive) unhealthy food choices | How susceptible someone is to making unhealthy food choices. For example, feelings of stress or an unhealthy environment can make you extra susceptible to (impulsively) choosing unhealthy food. | Professionals and citizens | Preference for fat/sugar/salt (+) | Focus on satiety rather than health of food (+)  Preference for fat/sugar/salt (+)  (Relative) budget healthy food (-)  Individual exposure unhealthy food (+)  Unclear/contradictory information health food (+)  Food skills/knowledge healthy eating (-)  Wanting to participate in unhealthy eating (+)  Interest/motivation healthy eating (-)  Priority healthy eating (-)  Tending towards convenience food  (+)  Capacity to act (-)  Unhealthy eating as compensation/masking (+)  Unhealthy eating as coping  (+)  Stress (+) |
| 60 | Skipping meals | Skipping meals means skipping a meal deliberately or out of necessity, for example due to lack of money. | Citizens | Hunger (+) | Lack of money (+)  (Relative) budget healthy eating (-) |
| 61 | Unhealthy eating as coping | Unhealthy eating as a way of dealing with stress or emotions. In stressful or sad situations, for example, you can seek comfort in unhealthy food. This is also called stress eating or emotional eating.  *Unhealthy eating as a coping mechanism can be (unconsciously) learned through your upbringing in which, for example, your parents started eating unhealthy food in response to stress and emotions.* | Citizens | Susceptibility (impulsive) unhealthy food choices(+) | Stress (+)  Upbringing healthy eating (-) |
| 62 | Unhealthy eating as compensation/  masking | Buying, sharing and/or eating unhealthy food in response to feelings of deficiency in other things. Out of shame (e.g. lack of money) parents may have their children hand out unhealthy treats to compensate for other things they feel they are deficient in, and to mask to others that they are not always able to keep up in things. | Citizens | Susceptibility (impulsive) unhealthy food choices(+) | Shame (+) |
